# Full-length transcriptome sequencing and comparative transcriptomic analysis to uncover genes involved in early gametogenesis in the gonads of Amur sturgeon (*Acipenser schrenckii*)

**DOI:** 10.1186/s12983-020-00355-z

**Published:** 2020-04-09

**Authors:** Xiujuan Zhang, Jiabin Zhou, Linmiao Li, Wenzhong Huang, Hafiz Ishfaq Ahmad, Huiming Li, Haiying Jiang, Jinping Chen

**Affiliations:** Guangdong Key Laboratory of Animal Conservation and Resource Utilization, Guangdong Public Laboratory of Wild Animal Conservation and Utilization, Guangdong Institute of Applied Biological Resources, Guangzhou, 510260 Guangdong China

**Keywords:** Amur sturgeon, *Acipenser schrenckii*, Isoform sequencing, Gonad transcriptome, Early gametogenesis

## Abstract

**Background:**

Sturgeons (Acipenseriformes) are polyploid chondrostean fish that constitute an important model species for studying development and evolution in vertebrates. To better understand the mechanisms of reproduction regulation in sturgeon, this study combined PacBio isoform sequencing (Iso-Seq) with Illumina short-read RNA-seq methods to discover full-length genes involved in early gametogenesis of the Amur sturgeon, *Acipenser schrenckii*.

**Results:**

A total of 50.04 G subread bases were generated from two SMRT cells, and herein 164,618 nonredundant full-length transcripts (unigenes) were produced with an average length of 2782 bp from gonad tissues (three testes and four ovaries) from seven 3-year-old *A. schrenckii* individuals. The number of ovary-specific expressed unigenes was greater than those of testis (19,716 vs. 3028), and completely different KEGG pathways were significantly enriched between the ovary-biased and testis-biased DEUs. Importantly, 60 early gametogenesis-related genes (involving 755 unigenes) were successfully identified, and exactly 50% (30/60) genes of those showed significantly differential expression in testes and ovaries. Among these, the Amh and Gsdf with testis-biased expression, and the Foxl2 and Cyp19a with ovary-biased expression strongly suggested the important regulatory roles in spermatogenesis and oogenesis of *A. schrenckii*, respectively. We also found the four novel Sox9 transcript variants, which increase the numbers of regulatory genes and imply function complexity in early gametogenesis. Finally, a total of 236,672 AS events (involving 36,522 unigenes) were detected, and 10,556 putative long noncoding RNAs (lncRNAs) and 4339 predicted transcript factors (TFs) were also respectively identified, which were all significantly associated with the early gametogenesis of *A. schrenckii*.

**Conclusions:**

Overall, our results provide new genetic resources of full-length transcription data and information as a genomic-level reference for sturgeon. Crucially, we explored the comprehensive genetic characteristics that differ between the testes and ovaries of *A. schrenckii* in the early gametogenesis stage, which could provide candidate genes and theoretical basis for further the mechanisms of reproduction regulation of sturgeon.

## Background

Sturgeons (Acipenseriformes) are polyploid chondrostean fish that originated during the Devonian period and have over 200 million years of history; thus, they constitute an important model species for studying development and evolution in vertebrates [1, 2]. As the source of caviar food, the sturgeon has high economic value, which has resulted in intense fishing pressure on wild stocks, so leading them to be listed among the more endangered group of species. In the updated February 2019 press release, the International Union for Conversation of Nature and Natural Resource (IUCN) Red List identified sturgeon as one of the most endangered animal groups; some 85% of sturgeon species are on the verge of extinction (http://www.iucnredlist.org/search). Efforts have been made worldwide to develop a sturgeon aquaculture industry for artificial reproduction since the early 1960s. However, to date, many new problems have emerged that require attention. For instance, The developmental asynchronism of the double sex gametes i.e. sperm and ovum and reproduction interval of 2–7 years markedly reduce reproduction efficiency in sturgeon artificial propagation practices, causing more aquaculture cost. Therefore, studies of gametogenesis mastering the mechanism of reproduction regulation in sturgeon are greatly useful for reproduction evaluation and aquaculture management.

Gametogenesis includes oogenesis and spermatogenesis, which mainly are comprised of germ cell growth and proliferation, primary spermatocytes and primary oocytes formation, until matured gametes of double sexes production. In fish, the onset of meiosis of germ cell is one of the most important steps in gametogenesis, which involved in the sex steroid hormones stimulating and the endocrine regulation [3, 4]. The previous studies reported various abnormalities in sturgeon gametogenesis and occurs of aberrant intersex gonads in cultured sturgeons [5, 6], which significantly impact on the viability of progeny and decrease the efficiency of sturgeon stock enhancement. In this context, the first and core step in investigating the key genes involved in early gametogenesis in sturgeon is to acquire full-length nucleotide sequences information of the genes. Whole-genome sequencing and assembly combined with transcriptome data would be an efficient way to systematically characterize gene models. However, the sturgeon genome is large and includes numerous mini-chromosomes and substantial polyploidy caused by genome duplication [7, 8], which possess significant difficulties to entire-genome sequencing. Recently, transcriptome-scale sex-related gene characterization was conducted in different sturgeon species with next-generation high-throughput sequencing technologies, including Adriatic sturgeon (*A. naccarii*) [9], Chinese sturgeon (*A. sinensis*) [10], Amur sturgeon (*A. schrenckii*) [11, 12] and Russian sturgeon (*A. gueldenstaedtii*) [13]. However, the genes acquired by only assemble procedure used in above studies signify incomplete sequences, which extremely restricts the yield of full-length genes. The third-generation long-read sequencing platform can overcome this difficulty.

In comparison with short-read sequencing, the methodological advantages of PacBio Isoform sequencing (Iso-Seq) include better completeness in sequencing both the 5′ and 3′ ends of the full-length cDNA molecules and greater accuracy in producing isoform-level transcripts. Recently, PacBio Iso-Seq technology has been successfully used in multiple species, such as *Populus* [14], Dolly Varden char [15], human cell lines and tissues [16], rabbits [17] and primates [18]. The transcriptomic data produced by PacBio Iso-Seq provide innovative research materials. For example, deciphering highly similar multigene family transcripts from Iso-Seq data with IsoCon has opened the door for gaining a deep understanding of genome evolution and human diseases [19], and the full-length transcripts from the Iso-Seq platform have provided new insight into the extreme metabolism of the ruby-throated hummingbird [20]. Meanwhile, the reconstruction and annotation of full-length transcripts also plays a critical role in gene discovery, particularly for species with no reference genome, such as the transcript variants involved in the innate immune system in *Litopenaeus vannamei* [21], gene families with two and more isoforms in *Misgurnus anguillicaudatus* [22] and transcript diversity in bioactive compound biosynthesis of *Astragalus membranaceus* [23]. Full-length transcriptome analyses may also drive new innovative progress for understanding the mechanisms of reproduction regulation in sturgeon.

In this study, we adopted joint PacBio Iso-Seq and short-read RNA-seq to generate a high-confidence full-length transcripts dataset of the gonads of 3-year-old *A. schrenckii* individuals and further used them to obtain comparative transcriptomic analysis for quantification investigation between testes and ovaries. Subsequently, a functional annotation of these full-length transcripts was systematically conducted with well-curated databases. Alternative splicing (AS) events, long noncoding RNAs (LncRNAs) and transcription factors (TFs) were detected. Most importantly, searching for genes involved in early gametogenesis and association analyses of related factors and early gametogenesis were performed. Herein, we not only provide a valuable resource i.e. a comprehensive full-length transcript set for the genomic-level reference of sturgeon but also systematically characterize early gametogenesis related genes of *A. schrenckii* to further investigate the functions of molecules during gametogenesis in sturgeon.

## Materials and methods

### Samples collection and histological analysis

A total of ten 3-year-old healthy Amur sturgeon (five males and five females) were sampled from the Engineering and Technology Center of Sturgeon Breeding and Cultivation of the Chinese Academy of Fishery Sciences (Beijing, China) in this study. Before sampling, the experimental individuals were anesthetized with eugenol in water for 1–3 min according to the AVMA guidelines for use (2013 version). The gonads i.e. ovaries and testes were separately collected and treated. One part of each gonad tissues was preserved in Bouin’s fixation for histological procedures, and another part was immediately immersed into liquid nitrogen until total RNA extraction. The histological analysis of each gonad tissue was performed using hematoxylin and eosin (HE) staining.

### RNA extraction and quality evaluation

Total RNA was extracted from each gonad tissue (testis or ovary) using RNAiso reagent (Takara, Tokyo, Japan). To prevent genomic DNA contamination, RNA sample was treated to digest DNA using RNase-free DNase I during extraction of total RNA. RNA purity and concentration were checked using a Nanodrop 2000 spectrophotometer (Thermo Scientific). RNA integrity was assessed using an Agilent RNA 6000 Nano reagents part I Kit in an Agilent 2100 Bioanalyzer System (Agilent Technologies, CA, USA). The RNA quality criteria for the RNA samples were RIN ≥ 7.0 (RNA Integrity Number), and 1.8 < OD260/280 < 2.2. The qualified RNAs were used for further PacBio and Illumina library construction, respectively. All the sequencing operations were conducted at Biomarker Technologies CO., LTD (Beijing, China). Considering the relatively large size of the sturgeon genome and the available *A. schrenckii i*ndividuals (a presumed octaploid species), the standard of sequencing quantity (clean data) was set as follows: Pacbio Iso-Seq of the library was at least 40 G and Illumina short-read RNA-seq was greater than 20 G for each sample.

### PacBio library construction and sequencing

To construct the library for PacBio sequencing, the qualified RNA from seven tissues, including the three testes and four ovaries, were mixed in equal amounts. The mixed RNA sample was reverse-transcribed for mRNA using the SMARTer™ PCR cDNA Synthesis Kit. PCR amplification was performed using the KAPA HiFi HotStart PCR Kit. Then, the PCR product for the SMRTbell library was constructed using the SMRTbell template pre kit. The concentration of the SMRTbell library was measured using a Qubit 3.0 fluorometer with a Qubit™ 1X dsDNA HS Assay kit (Invitrogen, Carlsbad, USA). The quantified criteria of library quality were concentration > 10 ng/μl with dispersive but continuous distribution in the range of 1–10kbp. A total of 2.5 ng of the library was sequenced for each SMRT cell using the binding kit 2.1 from the PacBio Sequel platform, producing 20 h of movies. The sample information was first registered as BioProject with accession number PRJNA532819 and BioSample with accession number SAMN11415730. Subsequently, the subread sequence generated by the PacBio Iso-Seq platform was deposited into the NCBI Sequence Read Archive (SRA) with accession number SRR7453063.

### Error correction of PacBio Iso-Seq reads

According to PacBio’s protocol, the raw polymerase reads were first processed using SMRTlink 5.0 software. Briefly, after removing the SMRTbell™ adapter and the low-quality data, postfilter polymerase reads were obtained. The circular consensus sequence (CCS) was generated from the subreads BAM files, also known as the reads of insert (ROI). All the ROIs whose number of full passes were > 1 were further classified into full-length (FL) and non full-length (nFL) transcript sequences based on whether the 5′ primer, 3′ primer and poly A tail could be simultaneously observed. We employed a three-step strategy for error correction to improve the accuracy of the full-length transcripts produced by the PacBio Iso-Seq platform. First, the circle sequencing with > 1 pass provided an opportunity for ROI self-correction. Second, full-length, nonchimeric (FLNC) reads were subjected to nonredundant and clustering treatments by the ICE Quiver algorithm and to Arrow polishing with the nFL sequence, producing high-quality and polished full-length consensus sequences. Finally, these polished consensus sequences were further subjected to correction and redundancy removal with Illumina short reads using the Proovread tool and the CD-HIT program with a –c 0.99 parameter cutoff [24], respectively. The above three corrections resulted in nonredundant, nonchimeric, full-length unigenes (isoform level) with high accuracy for subsequent analyses.

### Illumina library construction and sequencing

The Illumina library was prepared using the NEBNext, Ultra™ RNA Library Prep Kit (E7530 L) for Illumina (NEB, USA). Briefly, polyadenylated RNA was isolated and randomly separated into fragments. First-strand cDNA was synthesized using random hexamer primers, followed by second-strand synthesis. The purification of the double-stranded cDNA was performed using VAHTSTM mRNA capture beads. The purified and repaired double-stranded cDNA fragments were selected by size in the range of 250 bp ~ 350 bp. The concentration and quality control of the Illumina library were measured using a Qubit 3.0 fluorometer with an ExKubit dsDNA HS Assay kit (Invitrogen, Carlsbad, USA) and a Qsep 400 fragment analyzer, respectively. The quantified criteria of the library quality were a concentration > 1 ng/μl in a range of 380 bp ~ 480 bp fragments. The Illumina libraries were finally sequenced on the Illumina HiSeq platform.

Raw short reads in FASTQ format were first processed through in-house Perl scripts; clean short reads were obtained by removing reads containing adapter and ploy-N, and with low quality from the raw short reads. Simultaneously, the Q30, GC content and sequence duplication level of the clean data were calculated. The clean short reads were then mapped to the PacBio reference sequence using Tophat2 tools. Only reads with perfect matches or only one mismatch were further analyzed.

### Functional annotation of unigenes

For comprehensive functional annotation, the unigenes were searched against the following seven databases using BLAST software (version 2.2.26) [25]: NR (NCBI nonredundant protein sequences) [26], COG (Cluster of Orthologous Groups of proteins) [27], Pfam (Protein family) [28], Swiss-Prot (A manually annotated and reviewed protein sequence database) [29], KEGG (Kyoto Encyclopedia of Genes and Genomes) [30], GO (Gene Ontology) [31] and eggNOG (Cluster of Orthologous Groups of proteins) [32]. The Diamond BLASTX methods [33] with an E-value < 1 × 10^− 5^ were analyzed in NR, COG, Swiss-Prot, Pfam and KEGG annotations.

### Quantification of unigene expression levels and differential expression analysis

Using the full-length transcripts yielded from the SMRT Iso-Seq analysis as reference sequences, the unigene expression levels between the ovaries and testes of *A. schrenckii* were further analyzed based on the short-reads datasets generated by the Illumina sequencing platform. The extracts of three testes and four ovaries from seven *A. schrenckii* individuals were separately used as examples of the analysis.

Quantifications of the unigene expression values from the Illumina reads of each sample were determined with RSEM using the default parameters [34]. Briefly, the clean data from the Illumina sequencing were mapped back onto the reference sequences, and the readcount values of the unigenes for each sample were obtained. To eliminate the effects of the sequencing depth and transcript length, all the readcounts were transformed into FPKM values (expected number of fragments per kilobase of transcript sequence per million base pairs sequenced). The 0.1 threshold of FPKM value is regarded as the expression criterion of the unigene in tested tissue (FPKM > 0.1) according to the method [35].

To detect the differentially expressed genes (DEGs), all the readcounts of each sample were first normalized into a standardized readcount using the edgeR package. Then, the differential expression analysis of testes and ovaries was performed using the EBSeq R package mode based on the negative binomial distribution from the biological replicates. The resulting false discovery rate (FDR) values were adjusted using the posterior probability of being DE (PPDE) approach. Herein, the |log2(Fold Change)| > 1 and FDR < 0.05 were used as the threshold for determining DEGs.

### Alternative splicing prediction and analysis

Based on the BLAST method [25], all the unigenes were used for pairwise alignment. Finally, BLAST alignments that met all three of the following criteria were considered products of candidate AS events [36]. Briefly, 1) the length of two unigenes was both greater than 1000 bp, and there were two high-scoring segment pairs (HSPs) in the alignment. 2) The AS gap between two aligned unigenes was greater than 100 bp and at least 100 bp from the 3′ end and 5′ end. 3) A 5 bp overlap could be tolerated.

### LncRNA prediction and analysis

Unigenes with a length of over 200 nt and having more than two exons were selected as lncRNA candidates. Then, four computational approaches were employed to further screen the protein-coding unigenes from the noncoding unigenes: 1) we performed the coding-noncoding-Index (CNCI) with default settings [37] to assess the coding potential; 2) we used the coding potential calculator (CPC) to search for the unigenes in the NCBI eukaryotic protein database with a score < 0 setting [38]; 3) we translated each transcript in all three possible frames and used the Pfam scan utility with default parameters to identify the occurrence of any of the known protein family domains documented in the Pfam database [39]; 4) we used the coding potential assessment tool (CPAT) to assess the putative protein-coding unigenes by calculating the Fickett and Hexamer scores based on the logistic regression model [40]. As a result, all the isoform transcripts with coding potential were filtered, and the intersecting unigenes without coding potential formed our candidate set of lncRNAs.

### Transcription factor prediction and further analysis

Transcription factor (TF)-related unigene sequences were predicted using the BLAST method with the AnimalTFDB database [41]. The HMG domain of SOX was further verified using the SMART mode (http://smart.emblheidelbergde/index2.cgi).

According to the lengths of the four nonredundant full-length Sox9 genes, we renamed them as Asc_Sox9–1, Asc_Sox9–2, Asc_Sox9–3 and Asc_Sox9–4, respectively. A neighbor-joining phylogenetic tree was reconstructed based on amino acid sequences using the MEGA 7.0 package with the following parameter settings: p-distance model and partial deletion treatment. Meanwhile, Sox2 from the zebrafish *Danio rerio* (accession number: BAE48583.1) was chosen as the out-group protein sequence.

## Results

### Histology characteristics from the gonads of 3-year-old *A. schrenckii*

**Fig. 1:**
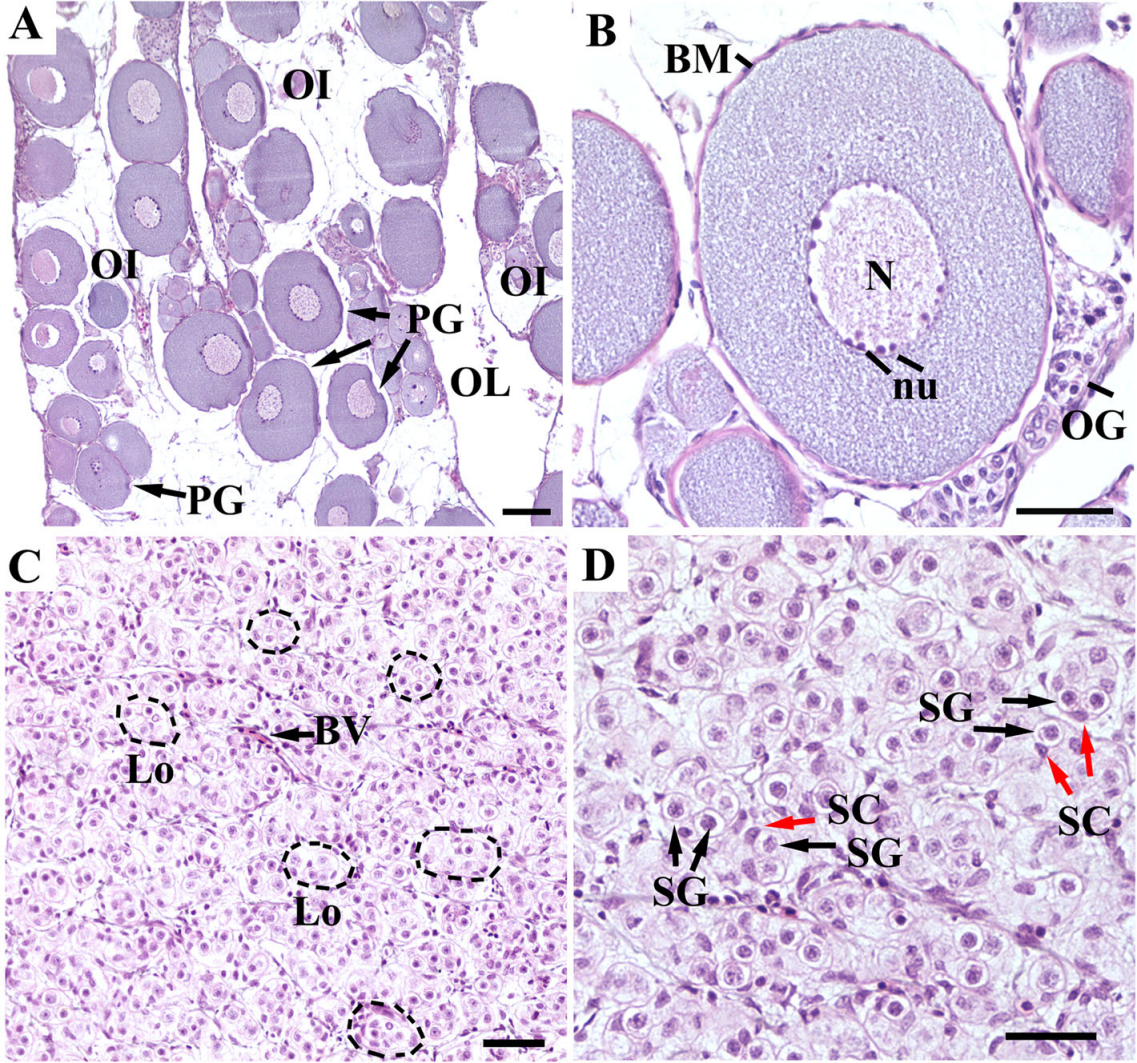
Histological characteristics of ovary (**a** and **b**) and testes (**c** and **d**) from 3-year-old *A. schrenckii* individuals. OI, ovarian lamellae; OL, ovarian lumen; PG, primary growth oocyte; BM, basement membrane; N, oocyte nucleus; Nu, oocyte nucleoli; OG, oogonia; BV, blood vessels; Lo, lobule; SG, spermatogonia; SC, Sertoli cell; The gonadal tissues were stained with hematoxylin and eosin (HE staining). Scale bar in A = 100 μm and in B, C, D = 50 μm

The gonads of 3-year-old *A. schrenckii* were visible at the early gametogenesis stage. In 3-year-old female individuals, the histological section of ovary showed deep, branching ovarian lamellae structure and was mainly composed of primary growth oocytes of different diameter sizes ranging from 100 to 500 μm. Some oocytes at the perinucleolar stage were clearly observed with a high number of small nucleoli along the nuclear perimeter. In 3-year-old male individuals, section of the entire germ region of testis showed smooth surface of the lateral side. By histological observation, it was found that the testis tissue displayed alveolate seminiferous lobules organization structure. The anastomosing tubules were separated from each other by thin layers of compact connective tissues and filled with spermatogonia enveloped by their own Sertoli cells. Meanwhile, we also found that there were some degenerating oocytes probably undergoing autophagy and some spermatogonia with pyknotic nuclei were probably apoptotic. Histomorphological characteristics of the ovary and testis from 3-year-old *A. schrenckii* individuals are shown in Fig. 1.

### Full-length transcripts from the gonads of *A. schrenckii*

**Fig. 2:**
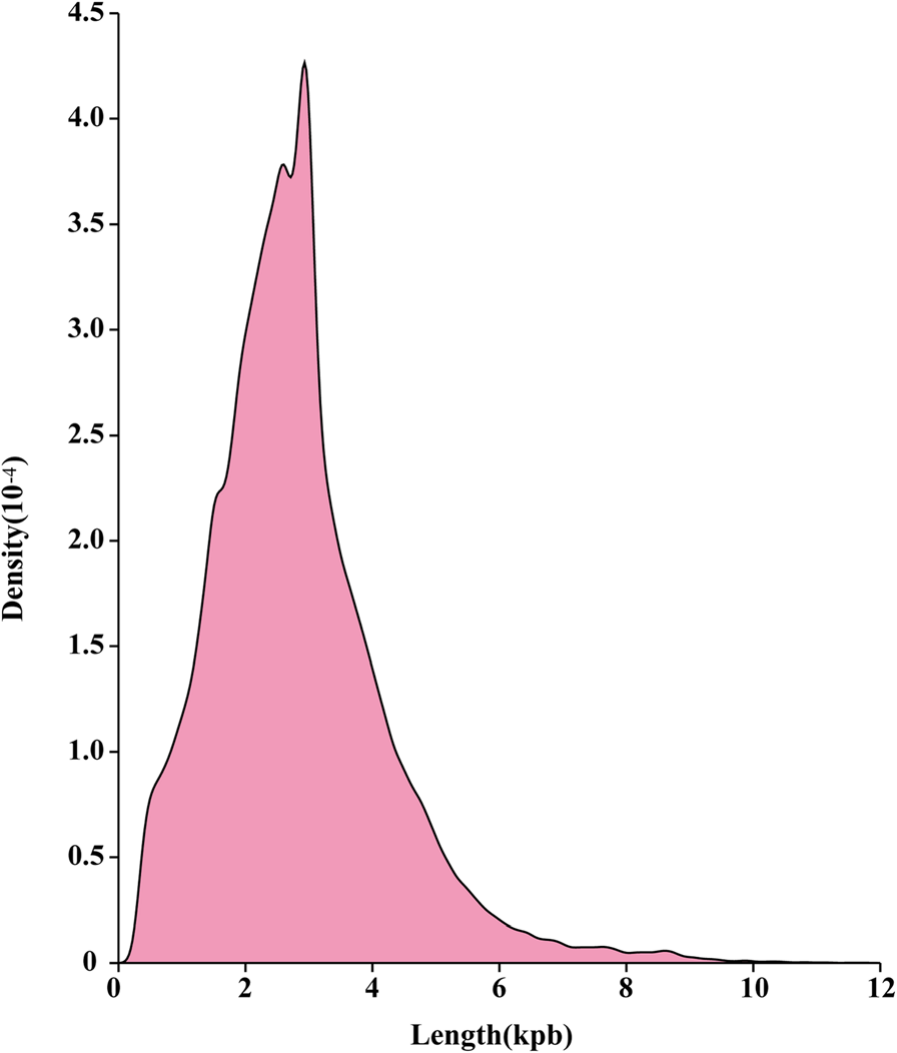
Distribution of nonredundant full-length unigenes from the gonad transcriptome of *A. schrenckii* by the PacBio Sequel platform

The full-length transcriptome of *A. schrenckii* was generated using the PacBio Sequel platform on the pooled RNA from seven tissues, including three testes and four ovaries. The resulting total of 50.04 G subread bases was generated by two SMRT cells from the PacBio library; therefore, the 1,260,958 reads of insert were produced with a mean read quality of 0.95 and mean passes of 14 circles (Supplementary Table 1). By applying the standard Iso-Seq classification and clustering protocol, all the ROIs were further classified into 358,153 nFL sequences and 860,617 FLNC reads with a mean length of 2548 bp. Based on the ICE Quiver and Arrow polishing algorithms, we produced 461,596 polished full-length consensus transcripts with a mean length of 2782 bp, including 335,067 high-quality (HQ) and 125,969 low-quality (LQ) sequences. After correction using short reads produced by Illumina short-read RNA-seq and subsequently removing redundancies using the CD-Hit program, the consensus transcripts were finally clustered into a total of 164,618 unigenes for subsequent analysis. We found that 91.09% of the unigenes main length distribution ranging from 1 to 6 kbp (Fig. 2 and Supplementary Table 2). The Iso-Seq statistics from the gonads of *A. schrenckii* by the PacBio Sequel platform are listed in Table 1.

**Table 1:** Description of Iso-Seq from the gonads of *A. schrenckii* by PacBio Sequel platform

species	SMRT cells	Subreads base (G)	Reads of Insert (ROIs)	nonfull-length reads	Full-length reads	FLNC reads	Consensus transcripts	Mean length (bp)	High-quality consensus transcripts	Low-quality consensus transcripts	Unigenes
*A. schrenckii*	2	50.04	1,260,958	358,153	869,918	860,617	461,596	2782	335,067	125,969	164,618

Meanwhile, the clean short reads were respectively generated from seven gonads of *A. schrenckii* (including three testes and four ovaries) using Illumina sequencing platform. The quality evaluation indexes of the clean short reads are summarized in Supplementary Table 3. In sum, the high-quality clean short reads from each sampled Illumina library was obtained with at least 6.96 × 10^7^ read numbers and a Q30 of over 90%.

### Efficient gene annotation of full-length unigenes from *A. schrenckii* gonads

To obtain a comprehensive functional annotation from the full-length gonad transcriptome of *A. schrenckii*, all the 164,618 nonredundant unigenes were assigned to align with seven different databases, including NR, KOG, Pfam, SwissProt, KEGG, GO, and eggNOG. A total of 93.55% of the unigenes (154,006 of 164,618) were successfully annotated with significant hits (E-value <1E^− 5^) from these well-curated databases. The statistics of the full-length unigene annotations are listed in Table 2. The remaining unannotated unigenes (10,612 unigenes) might represent novel *A. schrenckii* species-specific genes.

**Table 2:** Statistics of full-length unigene annotation performed with seven different databases

Database	NO. transcripts annotated	Annotated rate (%)
NO.unigene	164,618	–
NR	153,455	93.21
KOG	151,313	91.92
Pfam	134,181	81.51
SwissProt	102,427	62.22
KEGG	87,086	52.90
GO	72,758	44.20
eggNOG	47,463	28.83
Total	154,006	93.55

Among the annotated 60 classified GO terms, cellular process was identified as the most common annotation in the biological process; metabolic process and biological regulation were the next most abundant GO terms. In the molecular function and cellular component categories, binding and cell part annotations were identified as the most abundant terms, respectively. Two putative early gametogenesis related GO terms, including reproductive process (involving 1188 unigenes) and reproduction (involving 1134 unigenes), were successfully annotated. The GO classifications of the full-length unigenes from the gonads of *A. schrenckii* are shown in Supplementary Figure 1 and Supplementary Table 4.

In the KEGG classification, a total of 295 pathways annotated from 87,086 nonredundant unigenes were extracted from the gonad transcriptome of *A. schrenckii* (Supplementary Table 5). The results showed that the protein processing endoplasmic reticulum (2329 unigenes), RNA transport (2312 unigenes) and cell cycle (2263 unigenes) were the top three pathways with the most abundant unigenes. Notably, we paid attention to 14 KEGG pathways, which may be closely associated with early gametogenesis of sturgeon. Among these, MAPK signaling pathway (1834 unigenes), oocyte meiosis (1731 unigenes) and progesterone-mediated oocyte maturation (1398 unigenes) were the top three pathways with the most abundant unigenes distribution.

### Search for genes involved in early gametogenesis of *A. schrenckii*

A total of 60 genes (involving 755 unigenes), reported as active in the gonad development of sturgeon [10, 42–44], were detected to be present in the full-length gonad transcriptome of 3-year-old *A. schrenckii*. Therefore, the 60 early gametogenesis related genes and their NR annotations of full-length unigenes from the Iso-Seq of *A. scipenserkii* gonads are listed in Table 3 and Supplementary Table 6, respectively*.* Mainly, two major sex determination related genes in fish, Dmrt1and Gsdf, were also present in the full-length gonad transcriptome. Nine spermatogenesis-related genes were significantly matched with the full-length gonad transcriptome, including AR, Vasa, ER, Rspo1, Igf I, Dkk1, Hsd11b2, Cyp11b and ATRX. Meanwhile, four oogenesis-related genes were also predicted from the full-length gonad transcriptome, including Foxl2, Cyp19a, VR and Ozf. Four hormone receptor genes, including AR, ER, Gnrhr and Fshr. Two members of transforming growth factor (TGF)-β superfamily, Amh and Gsdf. Nine genes belonging to the sox subfamily, Sox3, Sox4, Sox5, Sox6, Sox7, Sox8, Sox9, Sox10 and Sox18, and five genes belonging to the double sex and mab-s (DM) domain, Dmrt1, Dmrt2, DmrtA1, DmrtA2 and DmrtB1, were found. In addition, Nine Sap family genes and four Hsp family genes were also concerned. We also found that Oct4 has the most abundant transcript diversity, with 100 unigenes predicted with ORFs among 104 unigenes, followed by Hsp70 (59/64) and Cyp19a (49/50).

**Table 3:** Searching for genes putatively involved in early gametogenesis from the full-length gonad transcriptome of *A. scipenserki*

Gene Symbol	Gene Description	Length (bp)	NO. Unigene	NO. Unigene with ORF
Dmrt1	double sex and mab-3 related transcription factor 1	2893&3843	2	0
Dmrt2	double sex and mab-3 related transcription factor 2	2291	1	1
DmrtA1	double sex and mab-3 related transcription factor A1	1422–2140	4	3
DmrtA2	double sex and mab-3 related transcription factor A2	2297–3868	12	12
DmrtB1	doublesex and mab-3 related transcription factor B1	2325–2377	4	0
Amh	anti-müllerian hormone	2071–3443	5	5
Foxl2	forkhead box protein L2	1802–2143	3	3
Bmp15	bone morphogenetic protein 15	1906–3389	11	7
Cyp19a	cytochrome P450 1A	1757–3305	50	49
Ctnnb1	catenin beta-1	3074–3598	13	13
OCT4	POU2	1830–4694	104	100
Lhx1	LIM homeobox 1	1213	1	1
Sf1	steroidogenic acute regulatory protein	2674	1	1
Rspo1	R spondin 1	2372	1	1
Sox3	SRY box 3	1335–3125	56	55
Sox4	SRY box 4	2257–3666	5	4
Sox5	SRY box 5	1500–6536	7	5
Sox6	SRY box 6	1491	1	0
Sox7	SRY box 7	1679–2211	15	13
Sox8	SRY box 8	2619–3152	7	7
Sox9	SRY-related HMG box gene 9	2657–3491	6	6
Sox10	SRY box 10	1968	1	1
Sox18	SRY box 18	2439	1	1
AR	androgen receptor	2941–8079	6	5
Vasa	vasa	2284–2876	30	27
ERɑ, ERβ	estrogen receptor	1956–6770	17	11
Pdgfrα	platelet-derived growth factor receptor alpha	3552	1	0
Hsd11b2	11-beta-hydroxysteroid dehydrogenase	2243–3278	3	2
Lhx9	LIM homeobox 9	3300&3332	2	2
Emx2	homeobox protein EMX2	2058	1	1
Fshr	follicle-stimulating hormone receptor	4201–5062	4	4
Gnrhr	gonadotropin-releasing hormone receptor	815&1002	2	0
Igf I	insulin-like growth factor I	2729&4884	2	2
Dkk1	dickkopf-related protein 1	803–1948	9	7
Wt1	wilms tumor protein homolog	3322	1	1
Fgfr2	fibroblast growth factor receptor 2	3916	1	1
VR	vitellogenin receptor	1224&3294	2	1
Fem1	fem-1 homolog A	1763–4084	13	10
Gsdf	gonadal soma derived factor	1602–2461	12	7
Spo11	meiotic recombination protein	1652–1797	5	3
Fstb2	follistatin b2	3181	1	1
Ozf6	oocyte zinc finger protein 6	1506–4396	47	35
Ozf7	oocyte zinc finger protein 7	1487–4252	21	17
Ozf22	oocyte zinc finger protein 22	3066	1	0
Hsp	heat shock protein	479–3651	7	7
Hsp70	heat shock protein 70	971–4279	64	59
Hsp75	heat shock protein 75	2289–2505	3	0
Hsp90	heat shock protein 90	403–7147	48	35
Kpi1	kunitz-type protease inhibitor 1	2541–4371	13	10
Gsf1	gametocyte-specific factor 1	592–6520	27	24
ATRX	ATRX	1417–8148	21	16
Sap2	spermatogenesis-associated protein 2	2141–4829	26	25
Sap5	spermatogenesis-associated protein 5	2419–3622	13	10
Sap6	spermatogenesis-associated protein 6	2173–4421	10	10
Sap7	spermatogenesis-associated protein 7	1666–3201	14	11
Sap13	spermatogenesis-associated protein 13	4707–6505	5	5
Sap17	spermatogenesis-associated protein 17	644–2371	3	2
Sap20	spermatogenesis-associated protein 20	3934	1	1
Sap22	spermatogenesis-associated protein 22	1406–1699	4	3
Sap24	spermatogenesis-associated protein 24	1691–5433	4	0

### Differential expression genes in early gametogenesis in the gonad transcriptome of *A. schrenckii*

**Fig. 3:**
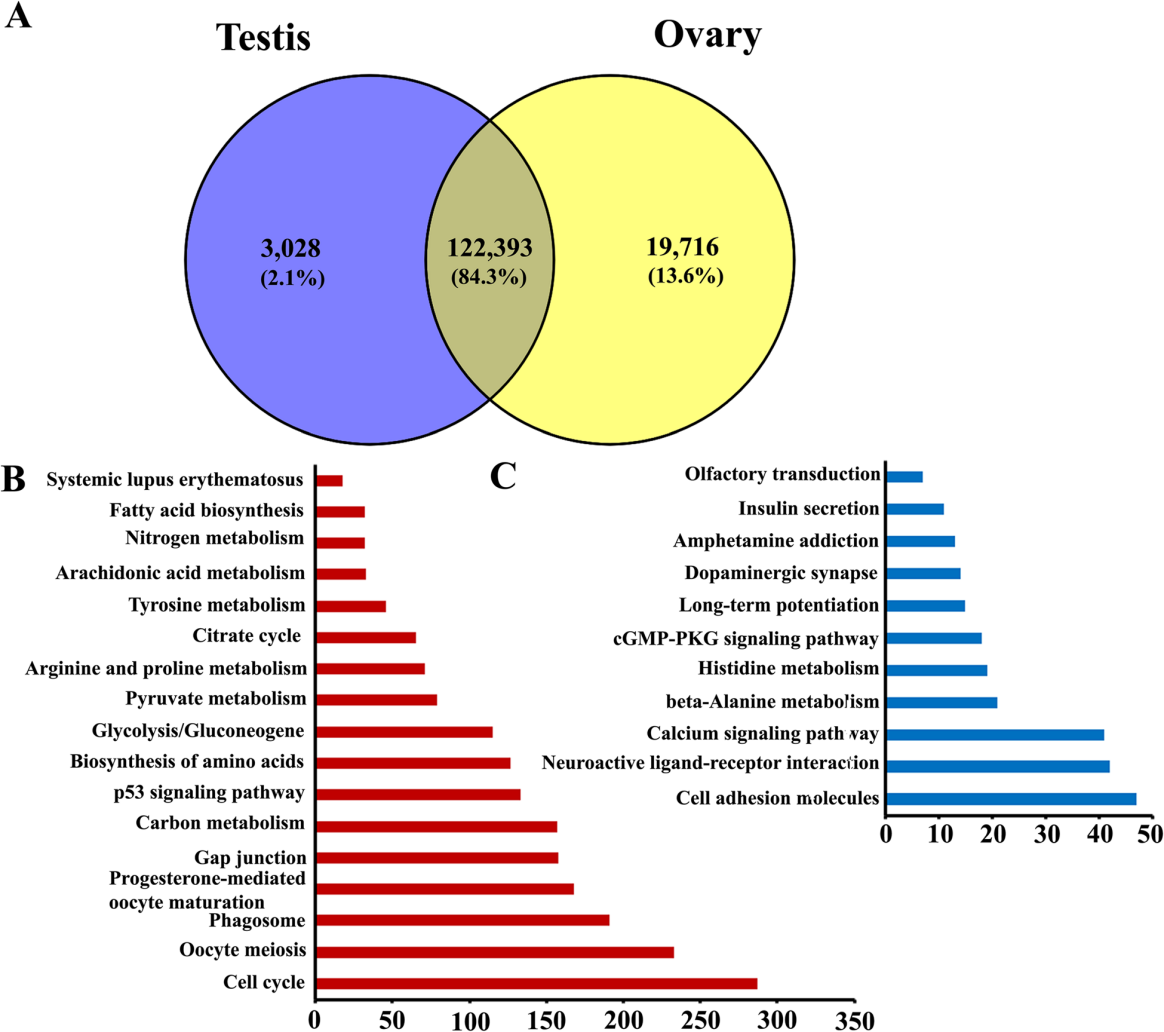
Difference analysis between the testes and ovaries of the full-length unigenes from the gonad transcriptome of *A. schrenckii* by the PacBio Sequel platform*.***a** Venn diagram showing the distribution of testis-specific and ovary-specific unigenes. **b** and **c** the significantly enriched KEGG pathways in ovary-biased and testis-biased DEUs, respectively (corrected *P* < 0.05)

Using nonredundant full-length transcripts as genome sequence references and combining the clean short reads datasets from the Illumina sequencing platform, the expression values (FPKM) of all 164,618 unigenes in three testes and four ovaries of seven *A. schrenckii* individuals were separately obtained. Therefore, the expression characteristics of all the full-length unigenes between the testes and ovaries of *A. schrenckii* were classified into the following three categories. 1) 19,481 unigenes were not expressed in either ovaries or testes (FPKM = 0 or FPKM ≤0.1); 2) 19,716 unigenes (13.6%) were ovary-specific expression patterns, including 14,284 unigenes in 0.1 < FPKM< 2, 4242 unigenes in 2 < FPKM< 10 and 1190 unigenes with FPKM> 10; 3) In contrast, only 3028 unigenes (2.1%) were exclusively transcribed in testis tissues, including 2751 unigenes in 0.1 < FPKM< 2, 229 unigenes in 2 < FPKM< 10 and 48 unigenes with FPKM> 10. Here, the distribution of testis-specific and ovary-specific unigenes are shown in a Venn diagram (Fig. 3a).

DEseq software was used for the analysis of differentially expressed unigenes (DEUs) in the testes and ovaries. Among 24,101 DEUs with a |log2(Fold Change)| > 1 and FDR < 0.05, 18,863 unigenes were upregulated in the ovaries, while 5238 unigenes were upregulated in the testes (Supplementary Figure 2). In further analysis 30 genes of the 60 early gametogenesis-related genes were screened to have significant expression between the testes and the ovaries (Table 4). In total, twelve genes (Foxl2, Cyp19a, OCT4, Sox3, Sox7, Bmp15, Dkk1, Gsf1, Hsp, Hsp70, Hsp90 and Sap24) were shown to be upregulated in the ovaries, while eighteen genes (DmrtB1, Amh, Sox4, Sox5, Sox8, Sox9, Vasa, Rspo1, ERβ, Gsdf, Hsd11b2, Fshr, ATRX, Ozf6, Ozf7, Sap2, Sap5 and Sap6) exhibited significant higher expression in the testes. Among the highly expressed genes in the testes, Amh is a only tissue-specific gene, i.e. only expressed in testes.

**Table 4:** Differential expression genes (DEGs) between ovary and testis of *A.schrenckii*

Unigene ID	Nr annotation ID	Gene symbol	Log_2_(OV/TE)^a^	FDR
**Relatively higher expression in ovary**				
F01_cb14613_c0/f2p1/2177	gi|742,229,200|ref.|XP_010900597.1|	Foxl2	4.52	0.0005
F01_cb22280_c14221/f1p1/3240	gi|348,162,225|gb|AEN19340.2|	Cyp19a	5.87	5.40E-07
F01_cb1470_c88/f1p1/2748	gi|341,579,644|gb|AEK81554.1|	OCT4	10.15	1.9E-05
F01_cb13001_c172/f9p26/1973	gi|573,890,381|ref.|XP_006632952.1|	Sox3	11.59	2.71E-12
F01_cb14611_c1/f1p0/2047	gi|573,875,677|ref.|XP_006626146.1|	Sox7	3.81	0.0027
F01_cb3737_c21/f1p0/1926	gi|573,890,192|ref.|XP_006632858.1|	Bmp15	4.75	4.3E-05
F01_cb15637_c23/f29p6/1372	gi|573,884,921|ref.|XP_006630539.1|	Dkk1	4.79	2.4E-05
F01_cb12452_c41/f1p0/1127	gi|742,184,514|ref.|XP_010888880.1|	Gsf1	4.08	0.0023
F01_cb8778_c103/f1p0/1710	gi|281,485,070|gb|ADA70351.1|	Hsp	3.69	0.0012
F01_cb13963_c503/f1p126/1797	gi|393,809,558|gb|AFM75819.2|	Hsp70	7.16	2.6E-06
F01_cb22281_c130670/f1p2/2775	gi|407,067,884|gb|AFS88930.1|	Hsp90	7.38	3.07E-08
F01_cb15971_c8/f2p1/1691	gi|632,957,530|ref.|XP_007894530.1|	Sap24	3.48	0.0067
**Relatively higher expression in testis**				
F01_cb7465_c17/f5p1/2348	gi|699,584,302|ref.|XP_009864039.1|	DmrtB1	−7.16	0.0069
F01_cb11611_c2106/f1p6/2113	gi|299,773,492|gb|ADJ38820.1|	Amh	inf ^b^	0.0035
F01_cb6093_c9/f1p0/2436	gi|51,599,123|gb|AAU08212.1|	Sox4	−4.33	0.0057
F01_cb1461_c13/f1p0/2264	gi|573,890,923|ref.|XP_006633221.1|	Sox5	−6.84	0.0002
F01_cb7554_c7/f1p0/2619	gi|410,586,767|gb|AFV74660.1|	Sox8	−4.39	0.0065
F01_cb5663_c3/f1p0/3843	gi|634,859,782|gb|AHZ62758.1|	Sox9	−5.38	0.0003
F01_cb6729_c44/f1p0/3330	gi|307,548,813|dbj|BAJ19133.1|	Vasa	−4.94	0.0053
F01_cb14310_c1/f1p0/2372	gi|573,887,629|ref.|XP_006631587.1|	Rspo1	−5.86	0.0008
F01_cb223_c11/f1p0/6792	gi|211,926,878|dbj|BAG82652.1|	ERβ	−4.55	0.0052
F01_cb11611_c593/f1p1/1932	gi|469,923,991|emb|CCP19133.1|	Gsdf	−8.82	2E-05
F01_cb11694_c1/f1p0/3278	gi|556,825,703|gb|AGZ80888.1|	hsd11b2	−6.49	0.0089
F01_cb10604_c20163/f1p0/4596	gi|573,902,226|ref.|XP_006638838.1|	Fshr	−7.03	1.3E-05
F01_cb10604_c21398/f1p1/4095	gi|573,889,889|ref.|XP_006632708.1|	ATRX	−4.87	0.0021
F01_cb10604_c55816/f1p3/3702	gi|742,250,963|ref.|XP_010867269.1|	Ozf6	−4.22	0.0021
F01_cb10604_c33459/f1p1/4252	gi|742,242,686|ref.|XP_010862827.1|	Ozf7	−4.77	0.0009
F01_cb22280_c10055/f2p1/3617	gi|573,907,277|ref.|XP_006641358.1|	Sap2	−4.11	0.0043
F01_cb22280_c4150/f1p0/3622	gi|573,881,115|ref.|XP_006628756.1|	Sap5	−6.48	0.0006
F01_cb22282_c89869/f1p0/2184	gi|573,883,855|ref.|XP_006630079.1|	Sap6	−4.09	0.0098

^a^The relative expression level of genes in ovary compared to that in testis. *OV* ovary, *TE* testis

^b^“inf” indicates tissue-specific expression pattern

To uncover the biological function of DEUs, we separately performed KEGG pathway enrichment analysis for the ovary-biased and testis-biased DEUs. As shown in Fig. 3b and c, we found that completely different KEGG pathways were enriched between the ovary-biased and testis-biased DEUs. A total of 17 and 11 terms significantly enriched from the ovary-biased and testis-biased DEUs were discovered, respectively (corrected *P* < 0.05). Among the ovary-biased DEUs, most of the unigenes were involved in the three top KEGG pathways, including the cell cycle, oocyte meiosis and phagosome. However, most of the unigenes of the testis-biased DEUs were related to cell adhesion molecules, neuroactive ligand-receptor interactions, calcium signaling pathways, etc. We further analyzed the distribution of DEUs in early gametogenesis-related GO terms and KEGG pathways. We found that oocyte meiosis is the most significant pathway with 277 DEUs of 1731 unigenes, followed by the progesterone-mediated oocyte maturation pathway with 201 DEUs (Table 5).

**Table 5:** DEUs of the early gametogenesis related GO terms and KEGG pathways in *A.schrenckii*

**GO classification**	**GO terms**	**NO.unigenes**	**DEUs**
Biological process	reproductive process	1188	143
Biological process	reproduction	1134	151
**KEGG pathway**	**Pathway ID**	**NO.unigenes**	**DEUs**
MAPK signaling pathway	ko04010	1834	153
Oocyte meiosis	ko04114	1731	277
Progesterone-mediated oocyte maturation	ko04914	1398	201
Wnt signaling pathway	ko04310	1298	123
TGF-beta signaling pathway	ko04350	768	108
GnRH signaling pathway	ko04912	712	67
Apoptosis	ko04210	563	45
Hedgehog signaling pathway	ko04340	399	40
Oxytocin signaling pathway	ko04921	203	18
Estrogen signaling pathway	ko04915	178	21
Steroid hormone biosynthesis	ko00140	150	12
Steroid biosynthesis	ko00100	119	13
Prolactin signaling pathway	ko04917	68	3
Ovarian steroidogenesis	ko04913	31	4

### Association analysis of alternative splicing (AS) and early gametogenesis from the gonad full-length transcriptomes of *A. schrenckii*

**Fig. 4:**
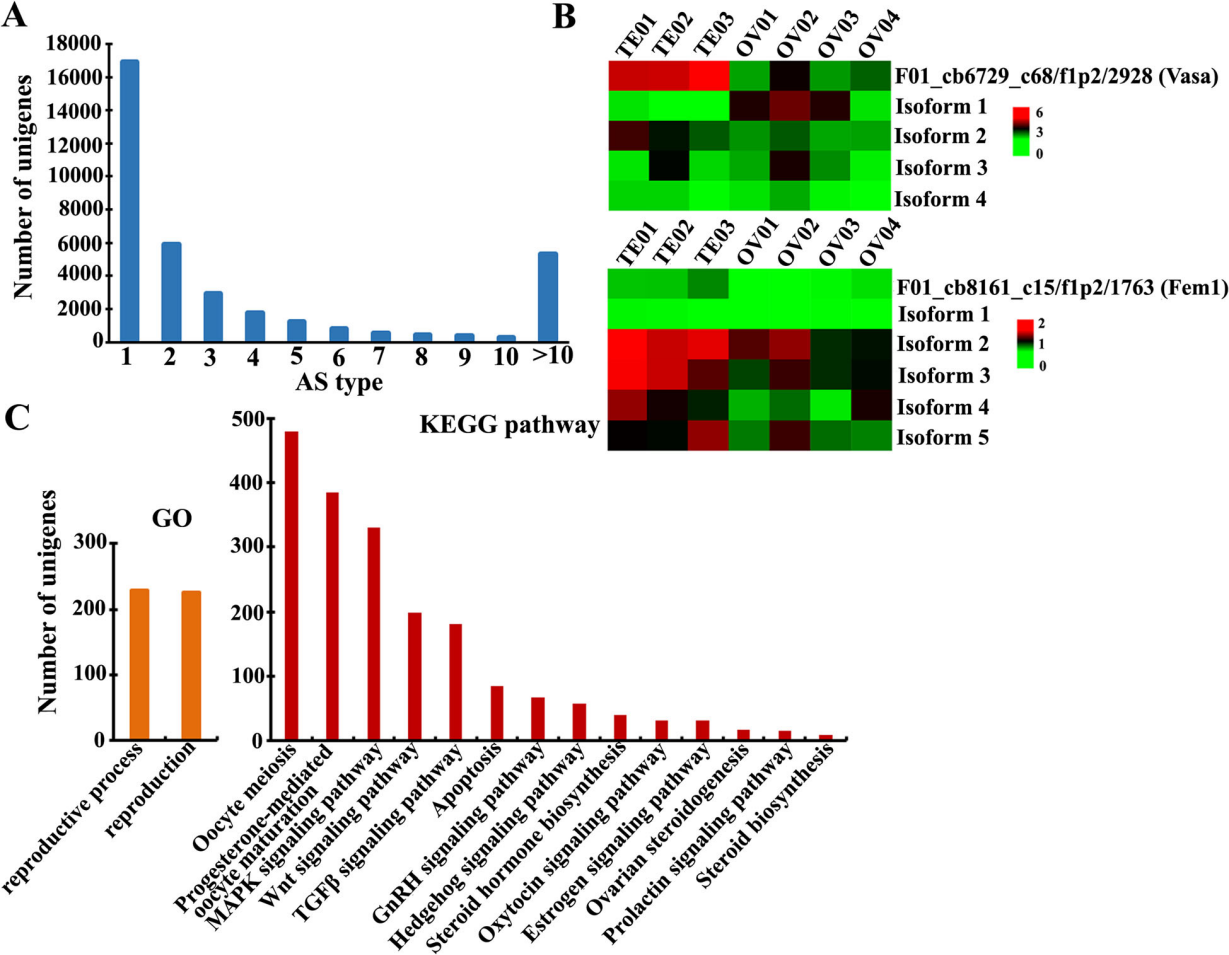
Alternative splicing (AS) analysis of the full-length gonad transcriptome of *A. schrenckii.***a** Statistics of the full-length unigenes detected with AS events. **b** The cluster heatmap (Log_2_(FPKM+ 1) values) indicates the expression patterns of different alternative isoforms in the testes and ovaries of *A. schrenckii.* Vasa (unigene ID: F01_cb6729_c68/f1p2/2928) predicted with four alternative isoforms and Fem1 (unigene ID: F01_cb8161_c15/f1p2/1763) with five alternative isoforms were selected as samples. **c** Distribution of AS events in early gametogenesis related GO terms and signaling pathways

Because sturgeon species still had no reference genome, we detected AS transcript isoforms of the full-length gonad transcriptome from *A. schrenckii* by referring to the pipeline of *Amborella trichopoda* without a reference genome [36]. As a result, a total of 236,672 AS events (involving 36,522 unigenes) were detected in the gonad transcriptome of *A. schrenckii*. Among these, we found that 16,909 unigenes (46.29%) had only one isoform; however, it is interesting to note that 5314 unigenes (14.55%) were predicted to have more than 10 isoforms (Fig. 4a).

Importantly, we found that sixteen early gametogenesis-related genes were predicted to be involved in AS events (Supplementary Table 7). We selected two genes as examples, including Vasa (unigene ID: F01_cb6729_c68/f1p2/2928) predicted with four alternative isoforms and Fem1 (unigene ID: F01_cb8161_c15/f1p2/1763) predicted with five alternative isoforms. In Fig. 4b, the cluster heatmap (Log_2_(FPKM+ 1) values) indicates the expression patterns of different alternative isoforms in the testes and ovaries of *A. schrenckii.* Vasa, as a exclusively expressed marker gene in germ-line, showed the predominant expression transcript with higher expression level in testes than that in ovary among 4 isoforms. Isoform 1 of Vasa had an obviously high expression in ovaries, and the other isoforms showed very low expression levels in both testes and ovaries. Meanwhile, we also found that expression levels of Fem and its five isoforms might seem like a paradoxical pattern. For example, isoform 2 and isoform 3 were the two main expression transcripts and had relatively high expression levels in both the ovaries and the testes, but isoform 4 and isoform 5 showed the opposite pattern between the ovaries and the testes. Therefore, these alternative isoforms suggest an important role in the regulation of gene expression through compensation or neutralization effects.

Subsequently, we investigated the distribution of AS events in the early gametogenesis-related GO terms and KEGG signaling pathways. The results indicated that the AS event is a pervasive feature involved in the early gametogenesis processes of sturgeon. The column plot showed that oocyte meiosis, progesterone-mediated oocyte maturation and the MAPK signaling pathway are the three most abundant AS events unigenes, while steroid biosynthesis is the least abundant event, with only eight unigenes (Fig. 4c).

### Association analysis of long noncoding RNAs (LncRNAs) and early gametogenesis from full-length gonad transcriptomes of *A. schrenckii*

**Fig. 5:**
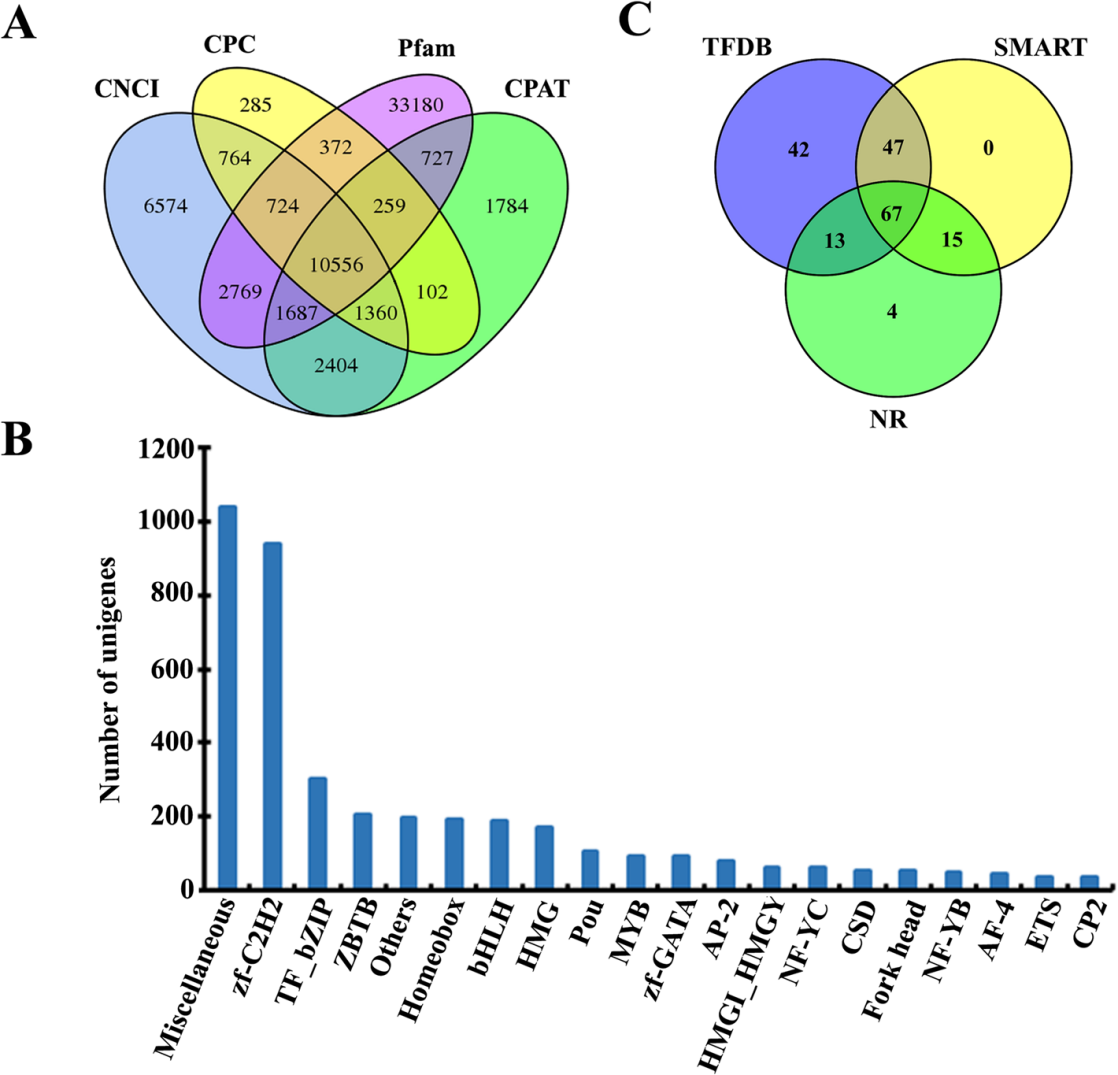
Identification of long noncoding RNAs (lncRNAs) and transcript factor (TF) analysis from the full-length gonad transcriptome of *A. schrenckii*. **a** Venn diagram of lncRNA prediction by four programs, including PLEK, CNCI, CPC and Pfam. **b** The top 20 abundant tems. **c** Transcript factor Sox family members were screened using AnimalTFDB alignment, SMART protein motif prediction and NR annotation

A total of 10,566 unigenes were identified as putative LncRNAs from the full-length gonad transcriptome of *A. schrenckii* (Fig. 5a)*.* Further analysis indicated that 2388 of the detected lncRNAs were not expressed in either the testes or ovaries (FPKM = 0 or FPKM≤0.1). Meanwhile, 524 LncRNAs present exclusively in ovary-specific expression and 551 LncRNAs present exclusively in testis-specific expression were also detected. Overall, 961 putative LncRNAs were differentially expressed between the ovaries and testes of *A. schrenckii,* including 235 ovary-biased LncRNAs and 726 testis-biased LncRNAs (Supplementary Figure 3).

### Association analysis of transcript factors (TFs) and early gametogenesis from full-length gonad transcriptomes of *A. schrenckii*

A total of 4339 nonredundant TF-related unigene sequences were matched against the AnimalTFDB database using BLAST, corresponding to 53 TF-related Pfam family domains. The top 20 abundant terms are listed in Fig. 5b. The sixty unigenes were found to have two or more different TF-related domains. For example, the results indicated that F01_cb10567_c40/f1p0/2683 was predicted to have a Homebox and miscellaneous domain, which suggests that further identification needs to be performed.

Many SRY-related HMG (high-mobility group) box (Sox) transcription factors play an important role in male gonadal differentiation, spermatogenesis and gonadal function maintenance in vertebrate species. Because the SOX family often shares the conserved HMG box domain, members of the Sox family were herein identified from the full-length gonad transcriptome of *A. schrenckii*. After further validation using SMART protein motif analysis, 82 nonredundant unigenes were found to have both HMG domains and SOX NR annotations with significant matches. We also found that the sequence variations of Sox increased the numbers of unigenes, and the 82 nonredundant unigenes belonged to seven members of the SOX family, including Sox3 (52 unigenes), Sox4 (4 unigenes), Sox5 (5 unigenes), Sox7 (10 unigenes), Sox8 (6 unigenes), Sox9 (4 unigenes) and Sox10 (1 unigene) (Fig. 5c and Supplementary Table 8).

**Fig. 6:**
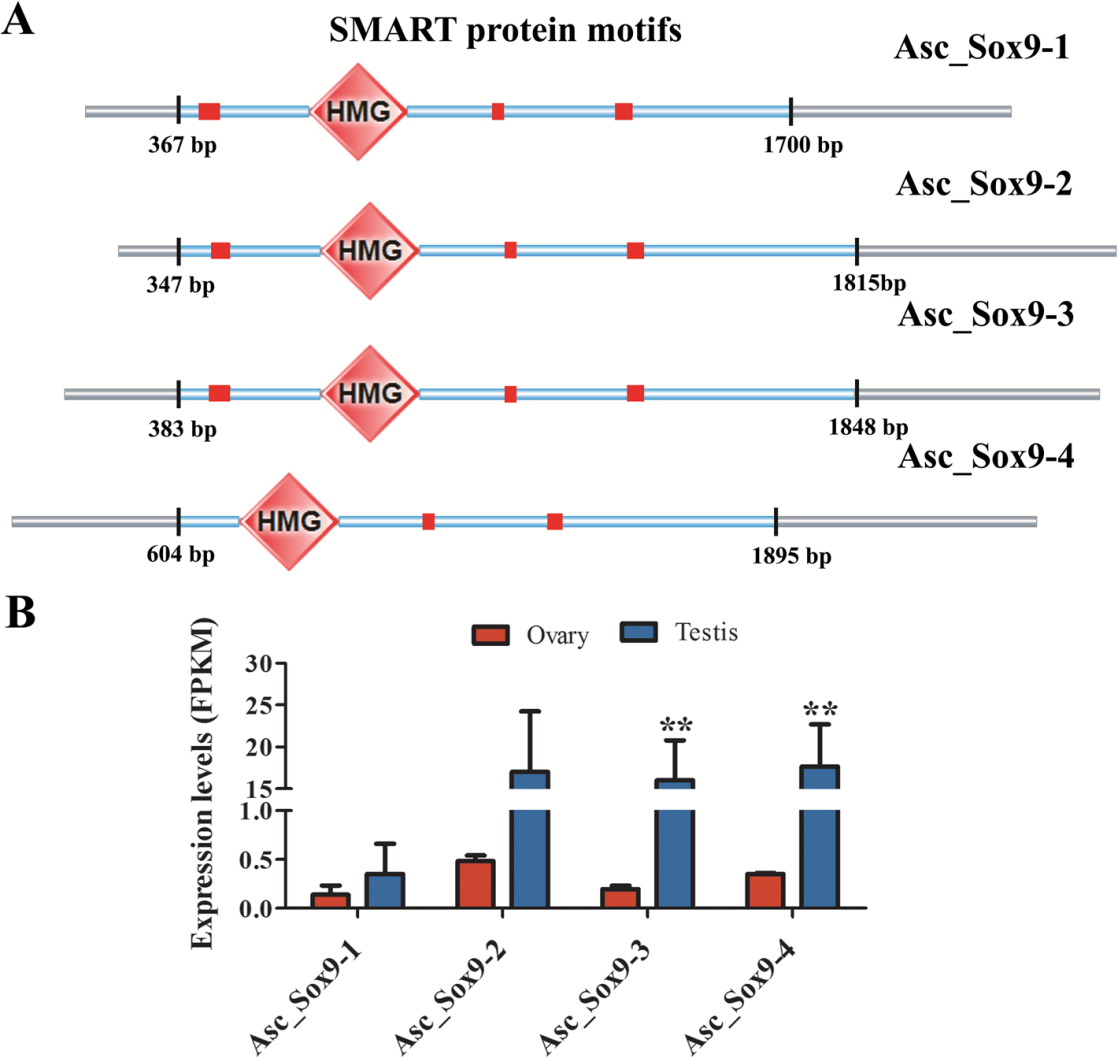
Gene structure of Asc_Sox9–1-4 and expression abundance (FKPM levels) in the testes and ovaries of *A. schrenckii*. **a** Achematic diagram of gene structure. The gray part indicates the UTR regions. The region between the black and vertical bars presents the SMART protein motifs. The diamond box shows conserved the HMG domain, and the red square indicates low complexity. **b** Expression abundance (FPKM levels) of Asc_Sox9–1-4 in testes and ovaries

Further analysis was performed using Sox9 as an example. The four nonredundant full-length unigenes were renamed Asc_Sox9–1-4 according to the length of PacBio Iso-Seq. The characteristics of the only completed sturgeon Sox9 protein from *A. sinensis* in the NCBI database (accession number: AHZ62758.1) and the Asc_Sox9–1-4 sequence are listed in detail in Table 6. The results showed two main differences: 1) compared to the length of Asi_Sox9 (2145 bp), those of the four Asc_Sox9–1-4 were longer and varied from 2873 bp to 3491 bp. 2) The lengths of the amino acids in Asc_Sox9–1-4 also varied, from 429 aa to 488 aa. The information for the Asc_Sox9–1-4 sequences is listed in Supplementary File 1. Due to the significant differences in amino acid lengths, HMG domain position, UTR length and expression abundance, the four Asc_Sox9 genes suggested putatively novel transcripts in *A. schrenckii*. Figure 6 shows a schematic diagram of the gene structure and expression abundance (FPKM levels) among the Asc_Sox9–1-4 genes.

**Table 6:** Characteristics of Sox9 gene sequences and their proteins

Name	Full-length (bp)	ORF (bp)	5’UTR (bp)	3’UTR (bp)	Amino acids (aa)	Molecular weight (kDa)	Isoelectric point
Asi_Sox9 (AHZ62758.1)	2145	1467	116	562	488	54.15	6.51
Asc_Sox9–1 (F01_cb5663_c14/f1p0/2873)	2873	1332	367	1174	443	48.98	6.58
Asc_Sox9–2 (F01_cb5663_c6/f1p0/3396)	3413	1467	347	1599	488	54.19	6.51
Asc_Sox9–3 (F01_cb5663_c3/f1p0/3843)	3429	1464	383	1582	487	53.94	6.48
Asc_Sox9–4 (F01_cb5663_c11/f1p0/3469)	3491	1290	604	1597	429	48.04	7.48

**Fig. 7:**
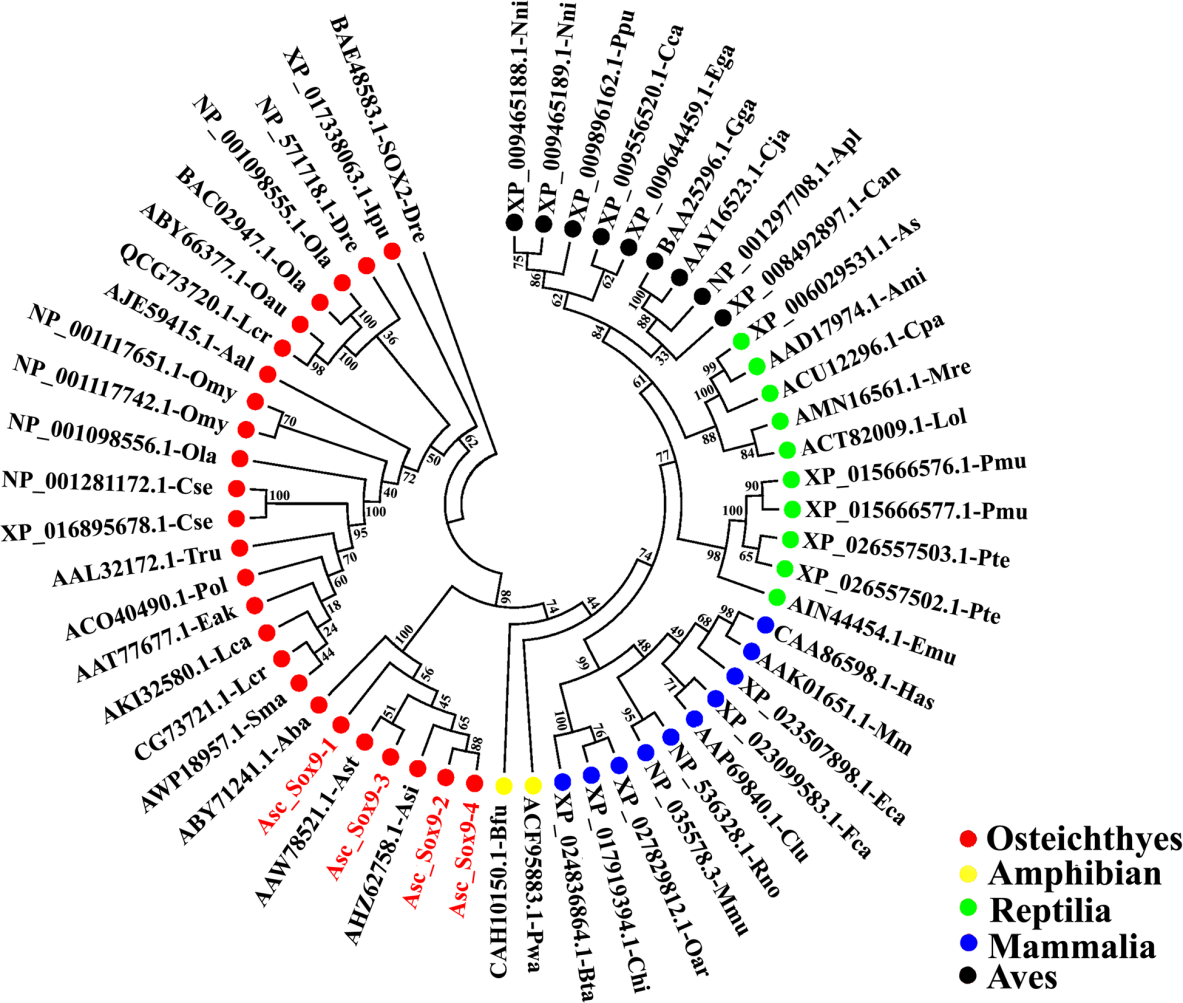
The phylogenetic tree of the Sox9 protein sequences was constructed using the neighbor-joining method. The node values represent the percent bootstrap confidence level derived from replicates. The accession numbers of the Sox9 proteins are shown in Supplementary Table 9. The five classes are comprised of Mammalia, Aves, amphibian, Reptilian and Osteichthyes. Meanwhile, Sox2 from zebrafish *Danio rerio* (accession number: BAE48583.1) was chosen as the out-group protein sequence

The phylogenetic tree was constructed from the alignment of the amino acid sequences in the four Asc_Sox9–1-4 genes with those from forty-six other animal species from five classes, including Mammalia, Aves, Amphibia, Reptilias and Osteichthyes (Supplementary Table 9). From Fig. 7, we found that all four Asc_Sox9–1-4 genes and those of other sturgeon species were perfectly clustered into one group. Obviously, Asc_Sox9–1 gene was closer to that of *A. baerii*; however, it was genetically distant from Asc_Sox9–3, Asc_Sox9–2 and Asc_Sox9–4. As expected, compared with the other four classes, the sturgeon Sox9 genes had the closest evolutionary relationship with other teleost fish species among all the five classes. We also found differences between the Sox9 gene tree and the species tree, which suggests that Sox9 is under evolutionary selection pressure. Meanwhile, Sox9 genes from five different classes were clustered together into five groups, which indicates that their functions may be relatively conserved in vertebrates.

## Discussion

Female sturgeons are more valuable than males due to the valuable caviar produced by their ovaries; consequently identifying sturgeon sex as early as possible and mastering the regulation technology of female gonad development could reduce production costs for enterprises. However, we still have poor knowledge for the regulatory mechanisms of reproductive processes, for example sex determination and differentiation, gonad development and gametogenesis. Amur sturgeon (*A. schrenckii*) is a critically endangered fish in the Acipenseridae family distributed in the Amur river in China and Russia [45]. Artificial reproduction of *A. schrenckii* began in the 1930s in China, and it has been the most popular sturgeon aquaculture species since the early 1990s. Currently, *A. schrenckii* has been one of the dominant caviar productions of farmed sturgeons and the popular crossing parents for sturgeon aquaculture in China [46, 47]. Here, the study of searching for genes involved in early gametogenesis and regulation mechanisms of reproduction control of *A. schrenckii* can provide practice application and theory basis. In the present study, we first produced high confidence and full-length transcriptome data from two independent types of gonad tissues (three testes and four ovaries), and maximized transcript diversity using the PacBio Sequel sequencing approach. As expected, a large amount of transcriptome data was generated, including 164,618 unigenes with a mean length of 2782 bp, compared with previous transcriptome reports yielding only N50 values with fewer than 1300 bp in *A. naccarii* [9]*, A. sinensis* [10]*, and A. schrenckii* [42]. Here, a total of 154,006 (93.55%) out of 164,618 full-length unigenes were successfully annotated as known homologous genes using seven well-curated databases. Due to the absence of reference genomic information, the remaining 10,612 full-length unigenes may suggest putative novel genes in sturgeon.

The testes tissue of 3-year-old *A. schrenckii* individuals was active in the mitotic proliferation stage of spermatogonia. In teleost fish, R-spondin 1 (Rspo1) expression was upregulated just before meiotic initiation in both the ovary and testis during the early developmental stages and the deficiency of Rspo1 was intriguingly found to cause a delay in spermatogenesis in XY fish [48]. In human, two estrogen receptor β (ERβ) wild-type transcript variants suggested specific functions in spermatogenesis due to their expression mainly located in somatic cells and primary spermatocytes [49]. The 11-beta-hydroxysteroid dehydrogenase (Hsd11b2) was expressed higher in male than female of 2-year-old *A. ruthenus* [50]. ATRX protein was present in adult human and rat testis and was expressed in spermatogonia, early meiotic spermatocytes and somatic cells [51]. Many members of spermatogenesis associated protein family genes (Sap) are identified in testes and play important roles in the spermatogenesis process in vertebrates including fish, for example, Sap2 [52, 53], Sap4 [54], Sap6 [55], Sap22 [56], and so on. In our study, above mentioned genes were also identified to be sex-significant expression genes in testes, which suggest they may paly conserved role in the early spermatogenesis of sturgeon.

Morphological observations indicated that ovary tissue was active in the stage of ovarian follicles development, when it filled with growing oocytes with different diameter sizes and abundant nucleoli in 3-year-old *A. schrenckii.* The forkhead transcription factor (Foxl2) plays an essential role in early ovarian development, subsequent maintenance of female trait and reproduction function. The knockout of Foxl2 was reported to give rise to complete female to male sex reversal in mammals and teleost fish [57, 58]. In fish, Foxl2 may play an important role in ovarian differentiation by maintaining cyp19a (cytochrome P450 1A) expression and antagonizing the expression of Dmrt1(double sex and mab-3 related transcription factor 1) [59]. In the present study, foxl2 and cyp19a were sexual dimorphic expression pattern and significantly higher expressed in the ovaries compared to the testes. Considering their significant role in the ovary, it would be necessary to explore the function of foxl2 and cyp19a further in the *A. schrenckii*. As the markers of undifferentiated spermatogonia, the expression patterns of Oct4 and Sox3 were found to have differentially expressed in the ovaries than that in the testes of the *A. schrenckii* transcriptome. The similar patterns were also reported in the previous Chinese sturgeon [10], which maybe signify a novel function in sturgeon.

At the molecular level, functional assignment indicated that the abundant transcript numbers and DEGs were mainly distributed in directly ovary-related KEGG pathways, including oocyte meiosis, progesterone-mediated oocyte maturation, oxytocin signaling pathway, estrogen signaling pathway, prolactin signaling pathway and ovarian steroidogenesis. Above results may suggest that the gonads of 3-year-old female Amur sturgeon are still in the core development stage for launching reproduction-related function activity. In mammals, the TGF-β signaling pathway has been shown to play important roles in the development of both ovarian and testicular functions [60, 61]. Moreover, the identification of Amh and Gsdf genes indicated that the TGF-β signaling pathway played a critical role in gonadal differentiation of teleost fish [62, 63]. In the present study, Amh and Gsdf, the two TGF-β subfamily numbers, were more significantly expressed in the testis compared to the ovary; in particular, the Amh gene had a testis-specific expression pattern. Therefore, whether the TGF-β signaling pathway is involved in sturgeon gonadal differentiation and how it works are worth further investigation.

In mammalian *Mus musculus*, the transcription factor Dmrt1 is sufficient to determine male fate, subsequent testicular development and both intrinsic and extrinsic control of spermatogenesis [64, 65]. In zebrafish, Dmrt1 functions in male sex determination and testis development [66]. In this study, Dmrt1 expression levels were not found to be significantly different between the testes and ovaries of *A. schrenckii*. A similar result was also observed in Chinese sturgeon [10] and the starlet *A. ruthenus* [43]. Coincidentally, Dmrt1 was reported that it does not participate in the initial steps of gonad differentiation in Siberian sturgeon [67] or Russian sturgeon [13]. Recent study indicated that Dmrt1 proteins were abundantly expressed in spermatocytes and spermatids, but not in type A spermatogonia of the Japanese eel [68]. Therefore, Dmrt1 expression also may be the stage-specific manner due to the pre-meiotic testes of 3-years-old *A.schrenckii*. However, another DM domain gene, DmrtB1, was found to be differentially expressed in the testes and ovaries of Amur sturgeon. DmrtB1 has been reported to play a pivotal role in coordinating the transition between mitosis and meiosis in murine germ cells and has a relevant role in the entry of spermatogonia into meiosis in humans [69]. Thus, it would be worth investigating the special role that DmrtB1 plays during the spermatogenesis of Amur sturgeon.

Although sturgeons have Müllerian ducts, which different from the teleost fish, anti-müllerian hormone (Amh) is reported to be conserved in a wide range of fish species and has a possible regulatory interaction with sex steroids and gonadotropic hormones in gonad development in fishes [70]. For example, the expression of Amh started in the gonads before sex differentiation, and its levels surged in the differentiated testes in *Danio rerio* [71]. An Amh testis-specific expression pattern was also detected in this study, strengthening the specific expression pattern in differentiated testes of sturgeon such as *A. baeri* [67] and *A. ruthenus* [50]. In teleost fish, a high diversity of sex candidate genes has been reported, for example Gsdf in *Oryzias luzonensis* [63] and Amhy (a duplicated copy of Amh) in the Patagonian pejerrey (*Odontesthes hatcheri*) [62]. Recent studies have identified major candidate genes for sex determination and differentiation based on conserved molecular mechanisms in many developmental events for vertebrate taxa, but the results vary among different species of sturgeon.

For the early gametogenesis related genes, we compared our results with published studies of other sturgeons. We found two distinct characteristics among all the studies. First, two hormone genes, Gnrh and Fsh, were reported to be transcribed in the gonads of 3-year-old *A. sinensis* [10]. However, no similar hormone genes could be found in the present study, the transcriptome data from 6 m *A. naccarii* [9], or even in five-year-old *A. gueldenstaedtii* [13]*;* we only found their corresponding receptors. Fish gonadal development is regulated by the hypothalamo-pituitary-gonad (HPG) axis. In axis, gonadotropin-releasing hormone (GnRH) expression is generally restricted to hypothalamus and promotes the synthesis and release of FSH and LH. FSH and LH are expressed in the pituitary by gonadotropic ells and activate their receptors and stimulate synthesis of the various sex steroid hormones in the gonads to regulate gametogenesis. That may be the main cause of no their transcripts in gonadal tissues. Second, Sox family numbers were found widely, which suggests a universal regulatory role in sturgeon gonadal differentiation. In this study, we provided an additional 31 full-length cDNA sequences grouped into five novel Sox family numbers for sturgeon species, including Sox5 (7 unigenes), Sox7 (15 unigenes), Sox8 (7 unigenes), Sox10 (1 unigene) and Sox18 (1 unigene). Therefore, a total of 14 Sox family numbers have been identified thus far from transcriptomes of sturgeon gonads.

The transcription factor Sox9, which is both necessary and sufficient for male fate and hence the maintenance of testis function in mammals [72], was found to have significantly higher expression in the testes than that in the ovaries of 3-year-old Amur sturgeon. The Sox9 gene expression characteristics in the other sturgeon species can be summarized as follows. In Russian sturgeon, Sox9 was expressed at significantly higher levels in the gonads of 2-year-old and 5-year-old males [13]. A similar report was made for 4-year-old stellate sturgeon (*A. stellatus*) individuals [73] and Siberian sturgeon during the early sex differentiation period [67]. These previous studies may suggest that Sox9 may have a core role in the early spermatogenesis of Amur sturgeon; however, additional studies i.e. function confirmation are necessary to verify the role of Sox9. In our study, we also found four novel Sox9 transcript variants with significantly different UTR region sequence and length. To our knowledge, the UTR region can play regulatory roles in mRNA transcription and translation. For example, the eukaryotic 5′ UTR is critical for ribosome recruitment to the mRNA and to start codon choice and that it plays a major role in the control of translation efficiency and shaping the cellular proteome [74]. The sequence and length of 3’UTR region impact on mRNA expression by association with microRNA (miRNAs). MiRNAs are endogenous, small non-coding RNAs (19–25 nucleotides in length) that functions as post-transcriptional repressors of gene expression by binding to complementary sites of 3’UTR of target mRNA. MiRNAs participate in pervasive biological processes, including growth, development, tumorigenesis [75]. Recent advances have been reported gonadal miRNAs directly control the differential expression of many sex-related genes to be involved in gametogenesis [76]; for example, miR-124 was involved in regulating the fate of developing ovarian cells by preventing the expression of Sox9 in mice [77]. An other study also reported that miRNAs can control mRNA fate by compartmentalization based on the 3’UTR length in male germ cells [78]. The present study is the first to report the four novel Sox9 transcript variants in the gonads of *A. schrenckii*. Excitedly, the identification of gonadal miRNAs and their differential expression in testis and ovary of *A. schrenckii* were also reported [79]. Therefore, investigation combined the early gametogenesis related full-length genes with gonadal miRNAs may help us to further understand the roles of reproduction regulation mechanism in sturgeon.

Isoform identification from the PacBio Iso-Seq strategy is one of is extremely important advantages. When using short read RNA-Seq strategies, extensive alternative prediction is impractical and high variability of isoform expression quantification is impossible in sturgeon without a true genome reference. However, the PacBio Iso-Seq conveniently provides the ability to finding greater numbers of AS events in genes of many species, even those of reference-free species [23, 36]. From the full-length gonad transcriptome data, we detected a huge number (236,672, involving 36,522 nonredundant full-length unigenes) of AS events in *A. schrenckii*. We also revealed that these AS events are universal, involving early gametogenesis related genes and widely existing in early gametogenesis related GO terms and KEGG pathways, which most likely corresponds to the complexity of the early gametogenesis function. For example, Vasa was proven to be important role in animal reproduction. The absence of functional Vasa leads to sterility, but the penetrance of this phenotype vary by sex. In Drosophila, Vasa deficiency results in female-specific sterility [80]. In mice and zebrafish, Vasa mutants have led to male-specific sterility [81, 82]. However, the regulation role of Vasa in gametogenesis is still unclear and previous reports mainly rely on expression studies. Vasa expression is restricted to only in both the ovary and testis with sexually dimorphic expression pattern. During gametogenesis, the Vasa protein was dynamically expressed in differentiating germ cells at different stages in adult gonads of the anadromous fish [83]. In *A. dabrynaus*, Vasa mRNA and protein displayed mitotic and meiotic expression in females, and mainly showed mitotic expression in males [84]. In our study, Vasa expression in both ovary and testis with sexually dimorphic pattern was consolidated in *A. schrenckii*, which maybe suggest the potential role in gametogenesis of sturgeon. Meanwhile, Vasa had relatively higher expression levels in testis than that in ovary, which is contrary with the ovary-biased pattern in Japanses flounder [85]. Importantly, we also revealed the four isoform variants of Vasa, which may imply having different biology functions during sturgeon early gametogenesis. Recent evidences indicate that different splice variants of Vasa exist in many species, including bovine [86] and Chinese mitten crab [87]. The qPCR further analysis indicated that the splicing variants of Vasa had different relative proportions during bull spermatogenesis. As 3-year-old *A. schrenckill* individuals were in the mitotic proliferation stage of spermatogonia, the testis-biased expression of Vasa suggests its roles in spermatogenesis, and further studies are required to investigate.

In summary, we combined PacBio Iso-Seq with Illumina short-read sequencing methods to conduct a comprehensive transcriptome analysis of the gonads of the Amur sturgeon, *A. schrenckii*. This approach enabled the generation of full-length transcripts as well as related analysis, that is, efficient gene annotation, alternative splicing, long noncoding RNAs and transcript factors. More importantly, isoform variants and expression profiles survey of the early gametogenesis related molecules of *A. schrenckii* contributed to a comprehensive insight into the early gametogenesis process and the reproduction regulation mechanisms of Amur sturgeon. Therefore, our study provides a valuable resource—a comprehensive full-length transcript set for genomic-level reference—which is both interesting and worthy of further in-depth studies in sturgeon.

## Conclusions

The present study provides the new genetic resources of full-length gonad transcriptome data and comparative transcriptome information of the gonads of the Amur sturgeon (*A. schrenckii*), an importantly economic aquaculture sturgeon species. A total of 164,618 high-quality nonredundant full-length transcripts (unigenes) generated from 50.04 G subread bases were herein produced with an average length of 2782 bp, which represents a significant advance in sturgeon genetics. The study discovered the number of 60 full-length genes identified to be related to early gametogenesis, further out of the 30 genes showed differential expression in the testes and ovaries suggesting significant function in the early gametogenesis of sturgeon. Interestingly, Amh with testis-specific expression and Gsdf with significantly higher expression in testes than ovaries (fold change > 200) belong to two key numbers of TGF-β superfamily, which may play core regulatory roles in the spermatogenesis of *A. schrenckii*; while the foxl2 combining with Cyp19a imply significant regulatory role in the oogenesis of *A. schrenckii*. Meanwhile, the four Vasa isoforms and four novel Sox9 transcript variants also hint function complexity of early gametogenesis of *A. schrenckii*. Finally, a total of 236,672 AS events, the 10,556 putative lncRNAs and the 4339 predicted TFs were identified to be involved in biological process of early gametogenesis of *A. schrenckii*. In total, our results provide first full-length gonad transcription data and information as a genomic-level reference for sturgeon. These importantly provide candidate genes and theoretical basis for further exploration of reproduction regulation of sturgeon.

## Supplementary information

**Additional file 1: Supplementary Figure 1.** The GO classification of the unigenes in *A. schrenckii.*

**Additional file 2:Supplementary Figure 2.** Volcano plot showing all the diferentially expressed unigenes (DEUs) in the gonad full-length transcriptome of *A. schrenckii*. The 18,863 DEUs occur in ovary-biased patterns and the 5238 DEUs occur in testis-biased patterns.

**Additional file 3: Supplementary Figure 3.** Volcano plot showing the 961 putative LncRNAs differentially expressed between the ovaries and testes of *A. schrenckii,* including 235 ovary-biased LncRNAs and 726 testis-biased LncRNAs.

**Additional file 4: Supplementary Table 1.** Summary of the ROIs from two SMRT cells in the gonads of *A.schrenckii.*

**Additional file 5: Supplementary Table 2.** The length distribution of full-length unigenes in the gonads of *A. schrenckii* acquired using the PacBio Sequel platform.

**Additional file 6: Supplementary Table 3.** Statistics of the clean short reads respectively from the testes and ovaries of seven *A. schrenckii* individuals using Illumina short-read RNA sequencing.

**Additional file 7: Supplementary Table 4.** Detailed GO annotation classification information for the unigenes of *A. schrenckii*.

**Additional file 8: Supplementary Table 5.** Detailed KEGG pathway annotation classification information for the unigenes of *A. schrenckii*.

**Additional file 9: Supplementary Table 6.** Information concerning the full-length unigenes annotated into early gametogenesis related genes using the NR database.

**Additional file 10: Supplementary Table 7.** The alternative splicing (AS) events of nonredundant early gametogenesis related unigenes in the full-length gonad transcriptome of *A.schrenckii.*

**Additional file 11: Supplementary Table 8.** Sox family members identified by TFDB, NR and SMART association analyses.

**Additional file 12: Supplementary Table 9.** Species and accession numbers of Sox9 proteins used in the phylogenetic analysis.

**Additional file 13: Supplementary File 1.** The cDNA sequences of full-length transcripts from 4 *A. schrenckii* Sox9s (Asc_Sox9–1-4) are underlined, including the 5′-untranslated region (UTR), the 3′-UTR containing a poly (A) tail and the open reading frame underlined.

## Data Availability

All data generated or analyzed during this study are included in this published article and its additional files.

## Abbreviations

Iso-Seq: PacBio isoform sequencing
DEUs: Differential expression unigenes
lncRNAs: Long noncoding RNAs
*A. schrenckii*: *Acipenser schrenckii*
AS: Alternative splicing
TFs: Transcript factors
FL: Full-length
nFL: Non full-length
FLNC: Full-length, nonchimeric reads
NR: NCBI nonredundant protein sequences
COG: Cluster of Orthologous Groups of proteins
Pfam: Protein family
Swiss-Prot: A manually annotated and reviewed protein sequence database
KEGG: Kyoto Encyclopedia of Genes and Genomes
GO: Gene Ontology
eggnog: Cluster of Orthologous Groups of proteins
FPKM: Expected number of fragments per kilobase of transcript sequence per million base pairs sequenced
DEGs: Differentially expressed genes
FDR: False discovery rate
CNCI: Coding-noncoding-Index
CPC: Coding potential calculator
CPAT: Coding potential assessment tool
