# Supplementary figures and images for: Full-length transcriptome sequencing and comparative transcriptomic analysis to uncover genes involved in early gametogenesis in the gonads of Amur sturgeon (Acipenser schrenckii)
Source: Front Zool. 2020 Apr 9;17:11. doi: 10.1186/s12983-020-00355-z (PMC7147073; doi:10.1186/s12983-020-00355-z)

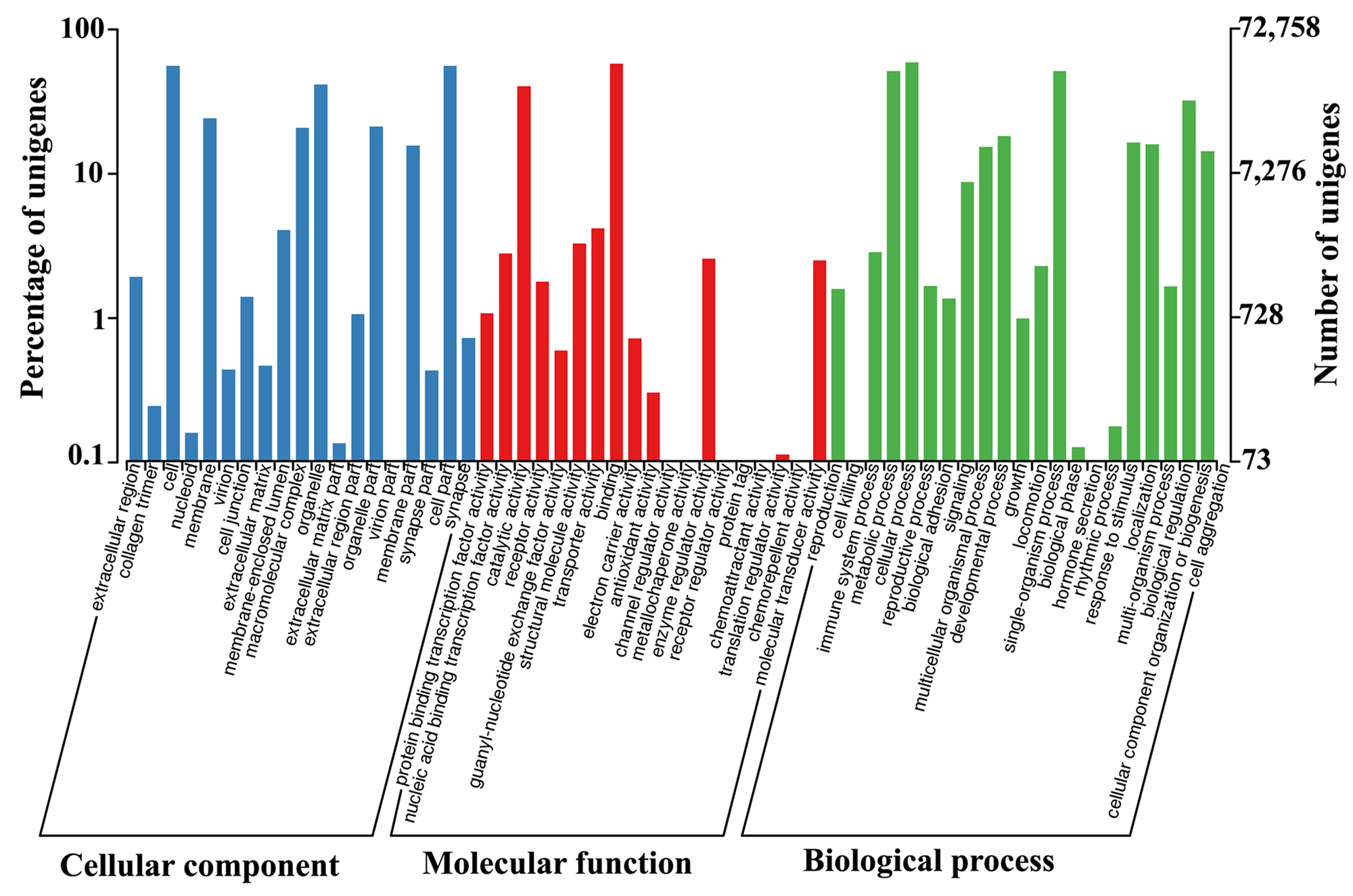

Supplement: Supplementary file 1 — Additional file 1: Supplementary Figure 1. The GO classification of the unigenes in A. schrenckii. [file 12983_2020_355_MOESM1_ESM.tif]

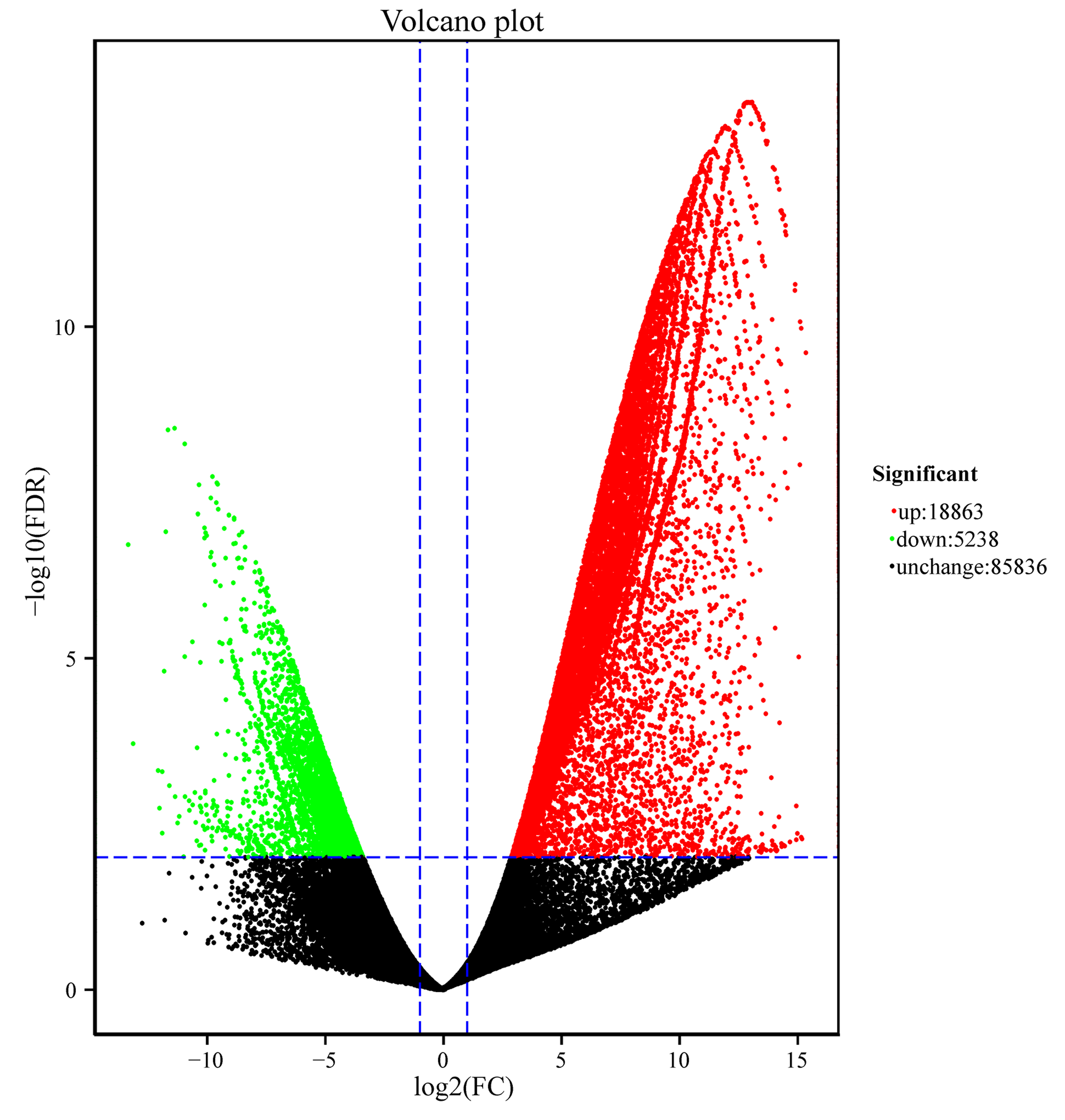

Supplement: Supplementary file 2 — Additional file 2:Supplementary Figure 2. Volcano plot showing all the diferentially expressed unigenes (DEUs) in the gonad full-length transcriptome of A. schrenckii. The 18,863 DEUs occur in ovary-biased patterns and the 5238 DEUs occur in testis-biased patterns. [file 12983_2020_355_MOESM2_ESM.tif]

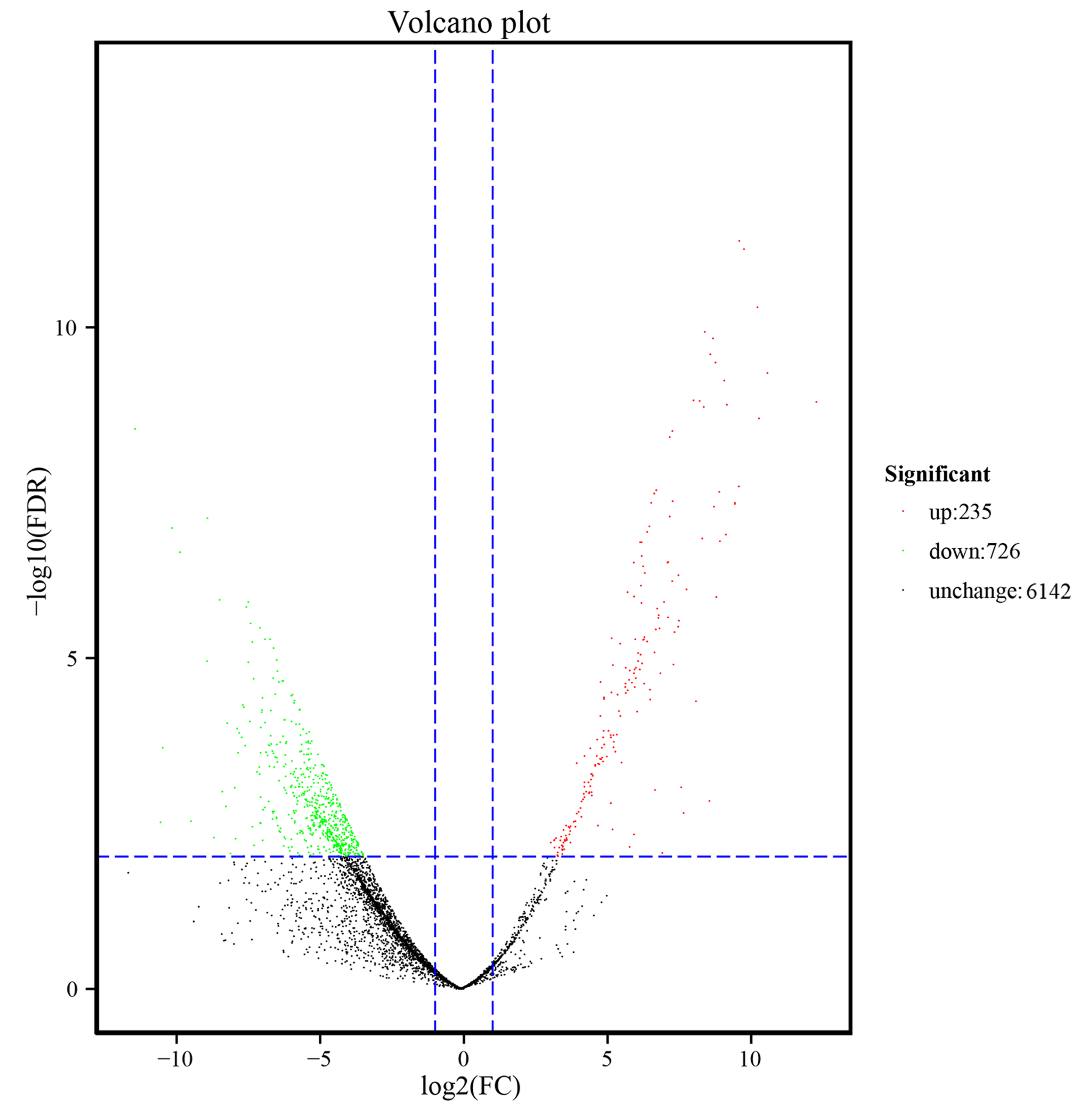

Supplement: Supplementary file 3 — Additional file 3: Supplementary Figure 3. Volcano plot showing the 961 putative LncRNAs differentially expressed between the ovaries and testes of A. schrenckii, including 235 ovary-biased LncRNAs and 726 testis-biased LncRNAs. [file 12983_2020_355_MOESM3_ESM.tif]
