# Supplementary material for: Full-length transcriptome sequencing and comparative transcriptomic analysis to uncover genes involved in early gametogenesis in the gonads of Amur sturgeon (Acipenser schrenckii)
Source: Front Zool. 2020 Apr 9;17:11. doi: 10.1186/s12983-020-00355-z (PMC7147073; doi:10.1186/s12983-020-00355-z)
Supplement: Supplementary file 13 — Additional file 13: Supplementary File 1. The cDNA sequences of full-length transcripts from 4 A. schrenckii Sox9s (Asc_Sox9–1-4) are underlined, including the 5′-untranslated region (UTR), the 3′-UTR containing a poly (A) tail and the open reading frame underlined. [file 12983_2020_355_MOESM13_ESM.docx]

**The cDNA sequences of full-length transcripts from four *A. schrenckii* Sox9s (Asc_Sox9-1-4) are underlined, including the 5’-untranslated region (UTR), the 3’-UTR containing a poly (A) tail and the open reading frame (ORF) underlined.**

**>F01_cb5663_c14/f1p0/2873 (Asc_Sox9-1)**

**cDNA:**

ACCTCAAAGCAGTTCAGTCCGTTTTGACATTTCCCTAAAGTAAACTGGAATAAAAATAAAAGGAACATTTTCTCTTTTATGCAAGACTAAATTGCACTTTTAAAGAAAACTGAATCGTGTATGCTTCGTCTACAAAAAATGCACATGCAATTCAACGATATTTGATACACAAGAACTTTATTTGCGGGTGTTTCGAAGAAAACTGTTTTTGTTGTTTTTTTAAAATCTCACCAAGGGACTTTGACAACTTCTTTGTATCCTCACTCAGCTCGAGATTGACCATTTAAGCCAAATCAGGCTAACTGTTTTTTTTTCTACTCACTTTCGTCGATTTACGATTTTCGTATGAATCTACTCGACCCCCTTC**ATGAAGATGACAGAGGACCAGGAGAAATCTCTGTCAGACGCCCCCAGCCCGAGCATGTCCGAGGATTCTGCGGGGTCCCCGTGCCCGTCTGGATCGGGCTCCGATGCTGAGAACACCAGACCGTCGGATAATAGCTTGTTGGGACCGGACAGTCAGATGCCGGACTTCAAAAAAGAGGGCGATGATGACAAATTCCCCGTTTGCATCAGGGATGCGGTTTCCCAGGTGCTGAAGGGCTATGACTGGACCCTGGTACCCATGCCCGTGCGGGTGAACGGAAGCAGCAAAAACAAGCCTCACGTTAAGAGACCAATGAATGCGTTCATGGTGTGGGCTCAGGCTGCCAGGAGAAAGCTGGCAGACCAGTACCCGCATCTTCACAACGCAGAACTCAGCAAAACACTCGGGAAACTTTGGAGATTGCTCAACGAAGGCGAGAAGCGTCCCTTCGTTGAAGAGGCAGAGAGACTGAGGGTGCAGCACAAGAAAGACCACCCCGATTACAAGTACCAGCCGAGGAGAAGGAAGTCTGTGAAGAACGGGCAGAATGAAGCTGAAGACGGATCTGAGCAAACTCACATCTCACCCACTGCGATCTTCAAAGCGCTGCAACAGGCCGATTCCCCTCATTCCGCATCCAGCATGAGCGAGGTGCATTCCCCCGGCGAGCATTCAGGCCAGTCCCAAGGTCCACCTACTCCGCCCACAACTCCCAAAACAGACATGCAGCCTGGAAAAGCAGATCTGAAGCGGGAAGGGCGCCCCCTCCAGGAAGGAGGTGGCAGGCAGCCTCACATTGACTTCAGAGATGTGGACATTGGAGAGCTGAGCAGCGACGTCATATCCAACATAGAGACATTCGATGTCAACGAGTTCGACCAGTACCTCCCGCCCAACGGTCACCCAGGTGTTCCAGCCACCCACTCAGCTCACGGCCAGAGTGGCCAGGTCACTTACACTGGCAGCTACGGCATCAGCAGCACCTCGGTCCCCCAAGCAGCAAATGTCGCGGGGCACGCTTGGATGGCCAAGCAGCAACAGCAGCAGCAGCAGCAGCACTCCCTGCCCACCCTCAGCAGCGAACAGGGGCAGCAGAGAACAACACACATCAAGACCGAGCAGCTGAGCCCGAGCCACTACAACGAGCAGCAGCACTCCCCGCAACCAATCAACCAGGGCTCCGGCCTGTACTCCACCTTCACCTACATGAGCCCGGCCCAGAGGCCCATGTACACCCCCATTGCAGACACTACCGGGGTCCCTTCCATCCCCCAGACCCACAGTCCTCAGCACTGGGAGCAGCCTGTCTACACACAGCTCACCAGGCCGTAG**GAGCCATTCAGACCGAATGCAGTGACTGTTGTGAGAAATGCCATCCCCATACATCTCAGAAACTGTTACTCTAGAGCCAGTGTGCCAGGATTCATTCAGTGTCAACTCAGATCTGAGCATGAACAACTTGAAACAGTGAACAAGAGCTGGGCTTTGGTCATTAAAAAGGCAGGTGAACTTTGAAGGGTTGGAATGGTTCAATCAGTGCCTTTTTGTGTTGAACGTAATAATTCATTGTTTTTTTTAGCCATAATTAAAAGAGAAATCCTCTGTGAGGACTTATTAATTCAAATATTTTTTAGTATGTACTGTGTATGTTTTATTCCTTTTTTTTGTTTTTTCGGGGGGATTTATACATATTTGTAGAGAAATGAAAGAAGCTCTTATTTTTTTTTCAAATCAGCTATATAAACCACTTGTGGTAGTACAGTCTGCTATAGCCTTTTTTGCTGACAAACATATTTTGTACAGAAAAAAAGAATTATATCTTTTTTTTTCCTCTGTCTCGCAATAATGTATTGTGCTTTTGTAACTTTGTAGCAAAAACAAAATGTTTGTAAAGAAAATGCTGGATAGTGTTCTAGTGATTAAAAAAAACAAAGTGCCAGTTATCAATGAAAGGGTTAAAAAAAAGAAAGATAGCTTTTGCATTCTTGGGCCTTACTACGTAAGTTAAAAAATAATGTATCATATGTTTTTTCTGCAAGACCTGTCTTTGCACCACTAGTGCCTATGGACCATGAATTCAGGAGCGAGATTAAATTTCCATGTTTTACTAGATTGACATCCACACCACACCAGCCCTGCACCAAGCCACAGCTGATATATTACTTCCTGTTTGACAGCATGTGCTAAACAGGCACAGGAAGTGACCTTTAACCCATTATGTTCCAGTAGGTCACAACAAAGTTACTTCAGTATTCCCCTCCCTATTCCTAACCCATTTAAAGTTATTTATTAGCATTAATTTATTTAAGTAAATATTTTCATTGTATTTGTTTTAATATACAGCTGTATATGACTAATTAAAAATTAAATAAATACCCTAGGTATATATATAAAAAAGCAGTTGATGGAAAGGGTATCCTTGTTGTTTTAATATGGTGCAAATTGTAACCATTGTTTATTTATCATTTTAAAGTTTCTGTTATTAAAAAAAGATTTTAAATCTTGT

**Protein:**

MKMTEDQEKSLSDAPSPSMSEDSAGSPCPSGSGSDAENTRPSDNSLLGPDSQMPDFKKEGDDDKFPVCIRDAVSQVLKGYDWTLVPMPVRVNGSSKNKPHVKRPMNAFMVWAQAARRKLADQYPHLHNAELSKTLGKLWRLLNEGEKRPFVEEAERLRVQHKKDHPDYKYQPRRRKSVKNGQNEAEDGSEQTHISPTAIFKALQQADSPHSASSMSEVHSPGEHSGQSQGPPTPPTTPKTDMQPGKADLKREGRPLQEGGGRQPHIDFRDVDIGELSSDVISNIETFDVNEFDQYLPPNGHPGVPATHSAHGQSGQVTYTGSYGISSTSVPQAANVAGHAWMAKQQQQQQQQHSLPTLSSEQGQQRTTHIKTEQLSPSHYNEQQHSPQPINQGSGLYSTFTYMSPAQRPMYTPIADTTGVPSIPQTHSPQHWEQPVYTQLTRP*

**>F01_cb5663_c6/f1p0/3396 (Asc_Sox9-2)**

**cDNA:**

ACTCAAAGCAGTTCGGTCCGTTTTGACATTTCCCTAAAGTAAACTGGAAATAAAAATAAAAGGAACATTTTCTCTTTTATGCAAGACTAACTTGCACTTTTAAAGAAAACTGACTCGTGTATGCTTCGTCTACAAAAAATGCACATGCAATTCAACGATATTTGATACACAAGAACTTTATTTGCGGGTGTTTCGAAGAAAACTGTTTTTGTTGTTTTTTTTAAATCTCACCAAGGGACTTTGACAACTTCTTTGTATCCTCACTCAGCTCGAGATTTGACCATTTAAGCCAAATCAGGCTAACTGTTTTTTTTTCTACTCACTTTCGTCGATTTACGATTTTTCGT**ATGAATCTACTCGACCCCTTCATGAAGATGACAGAGGACCAGGAGAAATCTCTGTCAGACGCCCCCAGCCCGAGCATGTCCGAGGATTCTGCGGGGTCCCCGTGCCCGTCTGGATCGGGCTCCGATGCTGAGAACACCAGACCGTCGGAGAATAGCTTGTTGGGACCGGACAGTCAGATGCCGGACTTCAAAAAAGAGGGCGATGATGACAAATTCCCCGTTTGCATCAGGGATGCGGTTTCCCAGGTGCTGAAGGGCTATGACTGGACCCTGGTACCCATGCCCGTGCGGGTGAACGGAAGCAGCAAAAACAAGCCTCACGTTAAGAGACCAATGAATGCGTTCATGGTGTGGGCTCAGGCTGCCAGGAGAAAGCTGGCAGACCAGTACCCGCATCTTCACAACGCAGAACTCAGCAAAACACTCGGGAAACTTTGGAGATTGCTCAACGAAGGCGAGAAGCGTCCCTTCGTTGAAGAGGCAGAGAGACTGAGGGTGCAGCACAAGAAAGACCACCCCGATTACAAGTACCAGCCGAGGAGAAGGAAGTCTGTGAAGAACGGGCAGAATGAAGCTGAAGACGGATCTGAGCAAACTCACATCTCACCCACTGCGATCTTCAAAGCGCTGCAACAGGCCGATTCCCCTCATTCCGCGTCCAGCATGAGCGAGGTGCATTCCCCCGGCGAGCATTCAGGCCAGTCCCAAGGTCCACCTACTCCGCCCACAACTCCCAAAACAGACATGCAGCCTGGAAAAGCAGATCTGAAGCGGGAAGGGCGCCCCCTCCAGGAAGGAGGTGGCAGGCAGCCTCACATTGACTTCAGAGATGTGGACATTGGAGAGCTGAGCAGCGACGTCATATCCAACATAGAGACATTCGATGTCAACGAGTTCGACCAGTACCTCCCGCCCAACGGTCACCCAGGTGTTCCAGCCACCCACTCAGCTCACGGCCAGAGTGGCCAGGTCACTTACACTGGCAGCTACGGCATCAGCAGCACCTCGGTCCCCCAAGCAGCAAATGTCGCGGGGCACGCTTGGATGGCCAAGCAGCAACAGCAGCAGCAGCAGCAGCACTCCCTGCCCACCCTCAGCAGCGAACAGGGGCAGCAGAGAACAACACACATCAAGACCGAGCAGCTGAGCCCGAGCCACTACAACGAGCAGCAGCACTCCCCGCAACCAATCAACTACGGCTCCTTCAACCTGCAGCACTACAGCTCTGCATATCCTACCATCACACGCTCCCAGTATGACTATTCAGAGCACCAGGGAGCCAACTCCTACTACAGCCACGCAGCAAACCAGGGCTCCGGCCTGTACTCCACCTTCACCTACATGAGCCCGGCCCAGAGGCCCATGTACACCCCCATTGCAGACACTACCGGGGTCCCTTCCATCCCCCAGACCCACAGTCCTCAGCACTGGGAGCAGCCTGTCTACACACAGCTCACCAGGCCGTAG**GAGCCATTCAGACCGAATGCAGTGACTGTTGTGAGAAATGCCATCCCCATACATCTCAGAAACTGTTACTCTAGAGCCAGTGTGCCAGGATTCATTCAGTGTCAACTCAGATCTGAGCATGAACAACTTGAAACAGTGAACAAGAGCTGGGCTTTGGTCATTAAAAAGGCAGGTGAACTTTGAAGGGTTGGAATGGTTCAATCAGTGCCTTTTTTTGTGTTGAACTTAATAATTAATTGTTTTTTTAGCCATAATTAAAAGAGAAATCCTCTGTGAGGACTTATTAATTCAAAATATTTTTTAGTATGTACTGTGTATGTTTTATTCCTTTTTTTTGTTTTTTTCGGGGGGATTTATACATATTTGTAGAGAAATGAAAGAAGCTCTTATTTTTTTTTCAAATCAGCTATATAAACCACTTGTGGTAGTACAGTCTGCTATAGCCTTTTTTTGCTGACAAACATATTTTTGTACAGAAAAAAAGAATTATATCTTTTTTTTTTCCTCTGTCTCGCAATAATGTATTGTGCTTTTGTAACTTTGTAGCAAAAACAAAATGTTTGTAAAGAAAATGCTGGATAGTGTTCTAGTGATTAAAAAAAACAAAGTGCCAGTTATCAATGAAAGGGTTAAAAAAAAAGAAAGATAGCTTTTGCATTCTTGGGCCTTACTACGTAAGTTAAAAAAATAATGTATCATATGTTTTTTCTGCAAGACCTGTCTTTGCACCACTAGTGCCTATGGACCATGAATTCAGGAGCGAGATTAAATTTCCATGTTTTACTAGATTGACATCCACACCATACCAGCCCTGCACCAAGCCACAGCTGATATATTACTTCCTGTTTGACAGCATGTGCTAAACAGGCACAGGAAGTGACCTTTAACCCATTATGTTCCAGTAGGTCACAACAAAGTTACTTCAGTATTCCCCTCCCTATTCCTAACCCATTTAAAGTTATTTATTAGCATTAATTTTATTTTAAAGTAAATATTTTCATTGTATTTGTTTTAATATACAGCTGTATACGACTAATTAAAAATTAAATAAATACCCTAGGTATATATATAAAAAAGCAGTTGATGGAAAGGGTATCCTTGTTGTTTTAATATGGTGCAAATTGTAACCATTGTTTATTTATCATTTTAAATGTTTCTGTTATTAAAAAAAGATTTTAAAATCTTGTATCTAGCTGTGTACAGATGTGTTTTAATTGTAATAAAACTTGATTAAAATTAGAAAAAAACAACAACTGGAAAAGTTCTTCTTAATTCTTAAAAACAAAAGTTGCAAATGTAGTGTATCACTGATTTGCATTTATATTTCCTGCTTCAGCTAAATCTTGGTAGTCAAGATTTTTAACTGTTTGGTTTTTTTAACTATGATCAAGCTGTGGCTGCATTGTAAAGACTTCTCTTATAAAAGACAGCATACTTTTTTTATTTTTCTTTTCTCTATTATATATCCCAGTACTGATTTTACAACTTTTTGTAATGCAACTGAACAAAACATATTGGGTTTTTTAGGAGTTTTAACAAATCGTCTTAAAAAGCTTATCTTTTGTATTATATTGTTTGCAATAAAAGAGAAAACGTATT

**Protein:**

MNLLDPFMKMTEDQEKSLSDAPSPSMSEDSAGSPCPSGSGSDAENTRPSENSLLGPDSQMPDFKKEGDDDKFPVCIRDAVSQVLKGYDWTLVPMPVRVNGSSKNKPHVKRPMNAFMVWAQAARRKLADQYPHLHNAELSKTLGKLWRLLNEGEKRPFVEEAERLRVQHKKDHPDYKYQPRRRKSVKNGQNEAEDGSEQTHISPTAIFKALQQADSPHSASSMSEVHSPGEHSGQSQGPPTPPTTPKTDMQPGKADLKREGRPLQEGGGRQPHIDFRDVDIGELSSDVISNIETFDVNEFDQYLPPNGHPGVPATHSAHGQSGQVTYTGSYGISSTSVPQAANVAGHAWMAKQQQQQQQQHSLPTLSSEQGQQRTTHIKTEQLSPSHYNEQQHSPQPINYGSFNLQHYSSAYPTITRSQYDYSEHQGANSYYSHAANQGSGLYSTFTYMSPAQRPMYTPIADTTGVPSIPQTHSPQHWEQPVYTQLTRP*

**>F01_cb5663_c3/f1p0/3843 (Asc_Sox9-3)**

**cDNA:**

ATGTCAAAGCAGTTCAGTCAGTCTTGACAGTTCCATAATAGAACCTGGAAATAAAAATAAAAGGAAAATTCATTTTTTTTTCTTTTGCAAGAGTAACTTGCACTTTTAAAGAAAACTGAATCGTGTATGCTTCGTCTCAAAAAAGGCACATGCAATTCAATGATATTTCGATATTTGATGCACAAGAACTTTATCTGCGGGTGTTTCGAAGAAAAACGTTGTTTTTTTTACAGCTTGATAAGGGACTTTGACAGCTTTATTTGCCTGAGCAAATACAGTTTTGCAACTTTTTTAGTGTCCCCAATCAGCTCGAGGTTTGACCATTTAGCCAAATCAGGCTAACTGTTTTTTCTACTCGCTTTCGTCGATTTACGATTTTTCTT**ATGAATCTACTCGACCCCTTCATGAAGATGACAGAGGACCAGGAGAAATGTCTGTCAGACGCCCCCAGTCCGAGCATGTCCGAGGATTCTGCGGGGTCCCCGTGCCCGTCTGGATCGGGCTCCGACGCTGAGAACACCAGACCGTCGGAGAATAGCTTGTTGGGACCGGACAGTCAGATGCCGGACTTCAAAAAAGAGGGCGATGATGACAAATTCCCCGTTTGCATCAGGGATGCGGTTTCCCAGGTGCTGAAGGGCTATGACTGGACCCTGGTACCCATGCCCGTGCGGGTGAACGGAAGCAGCAAAAACAAGCCTCACGTTAAGAGACCAATGAATGCGTTCATGGTGTGGGCTCAGGCTGCCAGGAGAAAGCTGGCAGACCAGTACCCGCATCTTCACAACGCAGAGCTCAGCAAAACACTCGGGAAACTTTGGAGATTGCTCAACGAAGGCGAGAAGCGTCCCTTCGTTGAAGAGGCAGAGAGACTGAGGGTGCAGCACAAGAAAGATCACCCCGATTACAAGTACCAGCCGAGGAGAAGGAAGTCAGTGAAGAACGGGCAGAACGAAGCTGAAGACGGATCTGAGCAAACTCACATCTCACCCACTGCGATCTTCAAAGCGCTGCAACAGGCCGATTCCCCTCACTCTGCGTCCAGCATGAGCGAGGTGCATTCCCCCGGCGAGCATTCAGGCCAGTCCCAAGGTCCACCTACTCCGCCCACAACTCCCAAAACAGACGTGCAGGCTGGAAAAGCAGATCTGAAACGGGAAGGGCGCCCCCTGCAGGAAGGTGGTGGCAGGCAGCCTCACATTGACTTCAGAGATGTGGACATTGGAGAGCTGAGCAGCGACGTCATATCCAACATAGAGACATTCGATGTCAACGAGTTCGACCAGTACCTCCCGCCCAACGGTCACCCAGGTGTTCCAGCCACCAACTCGGCTCACGGCCAGAGTGGCCAGGTCACTTACACTGGCAGCTACGGCATCAGCAGCACCTCGGTCCCCCAAGCAGCAAACGCCGCGGGGCACGCTTGGATGGCCAAGCAGCAGCAGCAGCAGCAGCAGCACTCCCTGCCCACCCTCAGCAGCGAACAGGGGCAGCAGAGAACAACACACATCAAGACCGAGCAGCTGAGCCCGAGCCACTACAACGAGCAGCAGCACTCCCCGCAACCAATCAACTACGGCTCCTTCAACCTGCAGCACTACAGCTCTGCATATCCTACCATCACACGCTCCCAGTATGACTATTCAGAGCACCAGGGAGCCAACTCCTACTACAGCCACGCAGCAAGCCAGGGCTCCGGCCTGTACTCCACCTTCACCTACATGAGCCCGGCCCAGAGGCCCATGTACACCCCCATTGCAGACACTACCGGGGTCCCTTCCATCCCCCAGACCCACAGTCCTCAGCACTGGGAGCAGCCTGTCTACACACAGCTCACCAGGCCTTAG**GAGCCATTCAGACCGAATGCAGTGACTGTTGTGAGAAAGGCCATCCCCATACATCTCAGAAACTTACTCTAGAGCCAGTGTGCCAGGTCAGTGTCAACTCAGATCTGAGAATGAACAACTCGAAACAGTGAACAAGAGCTGGGCTTTGGTCATTTAAAAAGGCAGGTGAACAGTCTTTGAGAGTTGGAATGGTTCAATCAGTGCCTTTTTTGTTGGACTTATAATTCATTGTATTTTTTAGCCATAATTAAAAGAGAAATCCTCTGTGAGGACTTATTAATTCTAAATATATTTTAGTATGTACTGTGTATGTTTCATTCTTTTTGGGGGGGGGGGAGGATTTATACATATTTGTAGGTAAATAAAAGACGCTCTTATTTTTTATTTTTTTTCAGATCAGCTCTATAAACCACTTGTGGTAGTACAGTCTGCTATAGCCTTTTTTTGCTGACAAACATATTTTTGTACAGAAAAAAATAATTATATATATATTTTTTTTTTACTCTCTCGCAATAGTGTATTGTGCTTTTGTAACTTTTTGTAGCAAAAACAAAATGTAAAGAAAATGCTGGATAATGTTCTAGTGATAAAAACAAAAAACAAAGTGCCAGTTATCAATGAAAGGGTTAAAAAAAAATGAAAAGGTAGCTTTTACATCCTTGGGCCTTACTACATAACTTAAAGAAATGTATCATATGTTTTTTCTGCAAGATGCTGTCTTTGCACCACTAGTGCCTATGGACCATGCATGCAGGAGCGAGATTAAATTTCCATGTTTTACTAGATTGACATCCACACCACACCAGCCCTGCACCAAGAAGCCACAGCTGATATACTACTTCCTGTTTGACAGCACGTGCTAAACAGGCACAGTAAGTGACCTTTAACCCATTAAGTTCCAGCAGGTCACAACAAAGTTGCTTTAGTATTCCCCTCCCTATTCCTAATCCATTTAAAGTTATTTATTAGCATTAATTTTATTTCAAAGTAAATATTTGCATTGTGTTTGTTTTAATATACAGCTGTATACAACTAATTAAATTTAATAAATACCCAAGGTTTATATAAAAAGGCAGTTGATGGAAAGGGTATCCTTGTTGTTTTAATATGGTGCAAATTGTAACCATTGTTTATTTATCATTTTAAGTGTTTCTGTTATTAAAAAAAATATTTTAAAGTCTTGTATCTAGCTGTGTACAGATGTGTTTTAATTGTAATTAAACGATTAAAATAAAAAAAAAACCAACTGGAAAAGTTATTTTTAATTTTTAAAAACAGAAGTTGCAAATGTAGTGTATCACTGATTTGCATTTATATTTCCTGCTTCAGCTTTGTTTTTTTAACTATGATCAAGCTGTGGCTGCTGTAAAGACTTCTCTTTATAAAAGACAAAATCCTTTTTTTTTTTTTTTAAAGTTCTATTTCTTTTCTCTATTATATATCCCAGTACTGATTTTTACAACTTTTTGTAATGCAACTGAACAAAACATATTGGGTTTTTTAGGAGTTTTAACAAATCATTGTCTTAAAAAGCTTATCTTTTGTATTATATTGTTTGCAATAAAAGAGAAAATAGCTAATT

**Protein:**

MNLLDPFMKMTEDQEKCLSDAPSPSMSEDSAGSPCPSGSGSDAENTRPSENSLLGPDSQMPDFKKEGDDDKFPVCIRDAVSQVLKGYDWTLVPMPVRVNGSSKNKPHVKRPMNAFMVWAQAARRKLADQYPHLHNAELSKTLGKLWRLLNEGEKRPFVEEAERLRVQHKKDHPDYKYQPRRRKSVKNGQNEAEDGSEQTHISPTAIFKALQQADSPHSASSMSEVHSPGEHSGQSQGPPTPPTTPKTDVQAGKADLKREGRPLQEGGGRQPHIDFRDVDIGELSSDVISNIETFDVNEFDQYLPPNGHPGVPATNSAHGQSGQVTYTGSYGISSTSVPQAANAAGHAWMAKQQQQQQQHSLPTLSSEQGQQRTTHIKTEQLSPSHYNEQQHSPQPINYGSFNLQHYSSAYPTITRSQYDYSEHQGANSYYSHAASQGSGLYSTFTYMSPAQRPMYTPIADTTGVPSIPQTHSPQHWEQPVYTQLTRP*

**>F01_cb5663_c11/f1p0/3469 (Asc_Sox9-4)**

**cDNA:**

ACTACAGACACTCAAAGCAGTTCGGTCCGTTTTGACATTTCCCTAAAGTAAACTGGAAATAAAAATAAAAGGAACATTTTCTCTTTTATGCAAGACTAACTTGCACTTTTAAAGAAAACTGACTCGTGTATGCTTCGTCTACAAAAAATGCACATGCAATTCAACGATATTTGATACACAAGAACTTTATTTGCGGGTGTTTCGAAGAAAACTGTTTTTGTTGTTTTTTTAAAATCTCACCAAGGGACTTTGACAACTTCTTTGTATCCTCACTCAGCTCGAGATTTGACCATTTAAGCCAAATCAGGCTAACTGTTTTTTTTTCTACTCACTTTCGTCGATTTACGATTTTTCGTATGAATCTACTCGACCCCTTCATGAAGATGACAGAGGACCAGGAGAAATCTCTGTCAGACGCCCCCAGCCCGAGCATGTCCGAGGATTCTGCGGGGTCCCCGTCCGTGGGGGGGGGGGGGGGGGGGGGGGGGGGGGTGGGGGGGGGGGGGGGGGGGGGGGGGGGGGGGGGGGGGGGGGTCCTGGATCGGGCTCCGATGCTGAGAACACCAGACCGTCGGAGAATAGCTTGTTGGGACCGGACAGTCAG**ATGCCGGACTTCAAAAAAGAGGGCGATGATGACAAATTCCCCGTTTGCATCAGGGATGCGGTTTCCCAGGTGCTGAAGGGCTATGACTGGACCCTGGTACCCATGCCCGTGCGGGTGAACGGAAGCAGCAAAAACAAGCCTCACGTTAAGAGACCAATGAATGCGTTCATGGTGTGGGCTCAGGCTGCCAGGAGAAAGCTGGCAGACCAGTACCCGCATCTTCACAACGCAGAACTCAGCAAAACACTCGGGAAACTTTGGAGATTGCTCAACGAAGGCGAGAAGCGTCCCTTCGTTGAAGAGGCAGAGAGACTGAGGGTGCAGCACAAGAAAGACCACCCCGATTACAAGTACCAGCCGAGGAGAAGGAAGTCTGTGAAGAACGGGCAGAATGAAGCTGAAGACGGATCTGAGCAAACTCACATCTCACCCACTGCGATCTTCAAAGCGCTGCAACAGGCCGATTCCCCTCATTCCGCGTCCAGCATGAGCGAGGTGCATTCCCCCGGCGAGCATTCAGGCCAGTCCCAAGGTCCACCTACTCCGCCCACAACTCCCAAAACAGACATGCAGCCTGGAAAAGCAGATCTGAAGCGGGAAGGGCGCCCCCTCCAGGAAGGAGGTGGCAGGCAGCCTCACATTGACTTCAGAGATGTGGACATTGGAGAGCTGAGCAGCGACGTCATATCCAACATAGAGACATTCGATGTCAACGAGTTCGACCAGTACCTCCCGCCCAACGGTCACCCAGGTGTTCCAGCCACCCACTCAGCTCACGGCCAGAGTGGCCAGGTCACTTACACTGGCAGCTACGGCATCAGCAGCACCTCGGTCCCCCAAGCAGCAAATGTCGCGGGGCACGCTTGGATGGCCAAGCAGCAACAGCAGCAGCAGCAGCAGCACTCCCTGCCCACCCTCAGCAGCGAACAGGGGCAGCAGAGAACAACACACATCAAGACCGAGCAGCTGAGCCCGAGCCACTACAACGAGCAGCAGCACTCCCCGCAACCAATCAACTACGGCTCCTTCAACCTGCAGCACTACAGCTCTGCATATCCTACCATCACACGCTCCCAGTATGACTATTCAGAGCACCAGGGAGCCAACTCCTACTACAGCCATGCAGCAAACCAGGGCTCCGGCCTGTACTCCACCTTCACCTACATGAGCCCGGCCCAGAGGCCCATGTACACCCCCATTGCAGACACTACCGGGGTCCCTTCCATCCCCCAGACCCACAGTCCTCAGCACTGGGAGCAGCCTGTCTACACACAGCTCACCAGGCCGTAG**GAGCCATTCAGACCGAATGCAGTGACTGTTGTGAGAAATGCCATCCCCATACATCTCAGAAACTGTTACTCTAGAGCCAGTGTGCCAGGATTCATTCAGTGTCAACTCAGATCTGAGCATGAACAACTTGAAACAGTGAACAAGAGCTGGGCTTTGGTCATTAAAAAGGCAGGTGAACTTTGAAGGGTTGGAATGGTTCAATCAGTGCCTTTTTTGTGTTGAACTTAATAATTCATTGTTTTTTTTAGCCATAATTAAAAGAGAAATCCTCTGTGAGGACTTATTAATTCAAAATATTTTTTAGTATGTACTGTGTATGTTTTATTCCTTTTTTTTGTTTTTTTCGGGGGGATTTATACATATTTGTAGAGAAATGAAAGAAGCTCTTATTTTTTTTTCAAATCAGCTATATAAACCACTTGTGGTAGTACAGTCTGCTATAGCCTTTTTTTGCTGACAAACATATTTTTGTACAGAAAAAAAGAATTATATCTTTTTTTTTCCTCTGTCTCGCAATAATGTATTGTGCTTTTGTAACTTTGTAGCAAAAACAAAATGTTTGTAAAGAAAATGCTGGATAGTGTTCTAGTGATTAAAAAAAACAAAGTGCCAGTTATCAATGAAAGGGTTAAAAAAAAAGAAAGATAGCTTTTGCATTCTTGGGCCTTACTACGTAAGTTAAAAAAATAATGTATCATATGTTTTTTCTGCAAGACCTGTCTTTGCACCACTAGTGCCTATGGACCATGAATTCAGGAGCGAGATTAAATTTCCATGTTTTACTAGATTGACATCCACACCACACCAGCCCTGCACCAAGCCACAGCTGATATATTACTTCCTGTTTGACAGCATGTGCTAAACAGGCACAGGAAGTGACCTTTAACCCATTATGTTCCAGTAGGTCACAACAAAGTTACTTCAGTATTCCCCTCCCTATTCCTAACCCATTTAAAGTTATTTATTAGCATTAATTTTATTTTAAAGTAAATATTTTCATTGTATTTGTTTTAATATACAGCTGTATACGACTTATTAAAAATTAAATAAATACCCTAGGTATATATATAAAAAAGCAGTTGATGGAAAGGGTATCCTTGTTGTTTTAATATGGTGCAAATTGTAACCATTGTTTATTTATCATTTTAAATGTTTCTGTTATTAAAAAAAGATTTTAAAATCTTGTATCTAGCTGTGTACAGATGTGTTTTAATTGTAATAAAACTTGATTAAAATTAGAAAAAAACAACAACTGGAAAAGTTCTTCTTAATTCTTAAAAACAAAAGTTGCAAATGTAGTGTATCACTGATTTGCATTTATATTTCCTGCTTCAGCTAAATCTTGGTAGTCAAGATTTTTAACTGTTTGGTTTTTTTAACTATGATCAAGCTGTGGCTGCATTGTAAAGACTTCTCTTATAAAAGACAGCATACTTTTTTTATTTTTCTTTTCTCTATTATATATCCCAGTACTGATTTTACAACTTTTTGTAATGCAACTGAACAAAACATATTGGGTTTTTTAGGAGTTTTAACAAATCGTCTTAAAAAGCTTATCTTTTGTATTATATTGTTTGCAATAAAAGAGAACGTAGTT

**Protein:**

MPDFKKEGDDDKFPVCIRDAVSQVLKGYDWTLVPMPVRVNGSSKNKPHVKRPMNAFMVWAQAARRKLADQYPHLHNAELSKTLGKLWRLLNEGEKRPFVEEAERLRVQHKKDHPDYKYQPRRRKSVKNGQNEAEDGSEQTHISPTAIFKALQQADSPHSASSMSEVHSPGEHSGQSQGPPTPPTTPKTDMQPGKADLKREGRPLQEGGGRQPHIDFRDVDIGELSSDVISNIETFDVNEFDQYLPPNGHPGVPATHSAHGQSGQVTYTGSYGISSTSVPQAANVAGHAWMAKQQQQQQQQHSLPTLSSEQGQQRTTHIKTEQLSPSHYNEQQHSPQPINYGSFNLQHYSSAYPTITRSQYDYSEHQGANSYYSHAANQGSGLYSTFTYMSPAQRPMYTPIADTTGVPSIPQTHSPQHWEQPVYTQLTRP*
